# *In vitro* methods in autophagy research: Applications in neurodegenerative diseases and mood disorders

**DOI:** 10.3389/fnmol.2023.1168948

**Published:** 2023-04-12

**Authors:** Dalinda Isabel Sánchez-Vidaña, Jingjing Li, Samuel Abokyi, Jackie Ngai-Man Chan, Shirley Pui-Ching Ngai, Benson Wui-Man Lau

**Affiliations:** ^1^Department of Rehabilitation Sciences, Faculty of Health and Social Sciences, The Hong Kong Polytechnic University, Kowloon, Hong Kong SAR, China; ^2^Mental Health Research Centre, The Hong Kong Polytechnic University, Kowloon, Hong Kong SAR, China; ^3^School of Optometry, Faculty of Health and Social Sciences, The Hong Kong Polytechnic University, Kowloon, Hong Kong SAR, China

**Keywords:** autophagy, cell-based assays, depression, neurodegenerative disorders, mood disorders

## Abstract

**Background:**

Autophagy is a conserved physiological intracellular mechanism responsible for the degradation and recycling of cytoplasmic constituents (e.g., damaged organelles, and protein aggregates) to maintain cell homeostasis. Aberrant autophagy has been observed in neurodegenerative diseases, including Alzheimer’s Disease (AD), Parkinson’s Disease (PD), Amyotrophic Lateral Sclerosis (ALS), and Huntington’s Disease (HD), and recently aberrant autophagy has been associated with mood disorders, such as depression. Several *in vitro* methods have been developed to study the complex and tightly regulated mechanisms of autophagy. *In vitro* methods applied to autophagy research are used to identify molecular key players involved in dysfunctional autophagy and to screen autophagy regulators with therapeutic applications in neurological diseases and mood disorders. Therefore, the aims of this narrative review are (1) to compile information on the cell-based methods used in autophagy research, (2) to discuss their application, and (3) to create a catalog of traditional and novel *in vitro* methods applied in neurodegenerative diseases and depression.

**Methods:**

Pubmed and Google Scholar were used to retrieve relevant *in vitro* studies on autophagy mechanisms in neurological diseases and depression using a combination of search terms per mechanism and disease (e.g., “macroautophagy” and “Alzheimer’s disease”). A total of 37 studies were included (14 in PD, 8 in AD, 5 in ALS, 5 in %, and 5 in depression).

**Results:**

A repertoire of traditional and novel approaches and techniques was compiled and discussed. The methods used in autophagy research focused on the mechanisms of macroautophagy, microautophagy, and chaperone-mediated autophagy. The *in vitro* tools presented in this review can be applied to explore pathophysiological mechanisms at a molecular level and to screen for potential therapeutic agents and their mechanism of action, which can be of great importance to understanding disease biology and potential therapeutic options in the context of neurodegenerative disorders and depression.

**Conclusion:**

This is the first review to compile, discuss, and provide a catalog of traditional and novel *in vitro* models applied to neurodegenerative disorders and depression.

## 1. Introduction

Autophagy is a vital intracellular machinery responsible for the clearance and recycling of cellular components (e.g., damaged organelles, protein aggregates, lipids, nucleic acids) (Bar-Yosef et al., 2019). Through this mechanism, the cytoplasmic cargo containing cellular waste is transported to the lysosome for degradation (Levine and Klionsky, 2017). Autophagy functions include maintaining cell homeostasis, supplying the cells with building blocks (e.g., amino acids, fatty acids) for the synthesis of new cellular components, supporting the generation of energy during cell renovation processes, and assisting cell growth and development (Mizushima et al., 2008; Mizushima and Komatsu, 2011; Park and Cuervo, 2013). Autophagy is a response mechanism to extracellular and intracellular stressors to drive the cell toward cell survival (Bar-Yosef et al., 2019; Nie et al., 2021). Autophagy is also part of the repertoire of programmed cell death mechanisms, the type II cell death mechanism, that is different from apoptosis (type I programmed cell death) (Nie et al., 2021).

Extracellular stress induced by nutrient deprivation, ischemia, and hypoxia leads to autophagy activation (Nie et al., 2021). Intracellularly, metabolic stress, and the presence of damaged organelles, unfolded proteins, or protein aggregates also activate the autophagy machinery (Ni et al., 2013; Saha et al., 2018). The mechanisms of autophagy are classified according to the mode of cargo delivery to the lysosome into macroautophagy, microautophagy, and chaperone-mediated autophagy (CMA) (Mizushima and Levine, 2020). Autophagy can also be categorized as selective autophagy in which specific cargo to be degraded is recognized, and non-selective autophagy in which random transport of organelles and other components to the lysosome occurs (Nie et al., 2021). Selective autophagy recognizes specific cargo (e.g., mitochondria, lipids, protein aggregates) for its degradation and can be categorized based on the type of cargo to be degraded (e.g., mitophagy, lipophagy, and aggregophagy) (Mizushima and Levine, 2020; Nie et al., 2021).

Macroautophagy has been extensively studied and is considered the major type of autophagy mechanism (Mizushima and Komatsu, 2011). A cytosolic double-membrane structure known as autophagosome is the intermediate organelle in macroautophagy and is formed from an isolation membrane known as phagophore (Mizushima and Komatsu, 2011; Bar-Yosef et al., 2019). The phagophore engulfs cytosolic components such as protein aggregates and damaged organelles for degradation and seals to form an autophagosome (Singh and Cuervo, 2011). The autophagosome travels along microtubules to reach the lysosome to fuse with it, becoming an autolysosome and starting the degradation and recycling of the cargo components through acidic lysosomal hydrolases (Mizushima and Komatsu, 2011; Bar-Yosef et al., 2019). An important parameter to measure in macroautophagy research is autophagy flux, defined as the dynamic process of autophagy. The evaluation of autophagy flux is essential in autophagy research as this process considers all the autophagy steps from autophagosome formation, maturation, fusion with lysosomes, and the release of catabolized components back to the cytosol (Zhang et al., 2013). Therefore, the evaluation of autophagy flux in *in vitro* models gives a complete picture of autophagy function as a dynamic and multistep process (Zhang et al., 2013). Another important concept in macroautophagy is lysosomal biogenesis (Tong et al., 2022). Lysosomal enzymes and lysosomal functions are pivotal in functional autophagy (Ding et al., 2022). The induction of lysosomal biogenesis has been suggested as a potentially effective therapeutic strategy (Ding et al., 2022). For instance, lysosomal acidity is normally reduced with aging which generates a suboptimal environment for the enzymatic activity in the catalytic process mediated by autophagy which has been seen to reduce the clearance of amyloid-β (AB) and tau aggregates in AD models (Tong et al., 2022).

In microautophagy, the lysosome itself engulfs cytoplasmic components by inward invagination of the lysosomal membrane that traps the cytosolic cargo and internalizes it for degradation (Singh and Cuervo, 2011; Mizushima and Levine, 2020). The invaginations of the lysosomal membrane form multivesicular bodies (MVB) (Sahu et al., 2011). After the cargo is wrapped around by the lysosomal membrane, the membrane is rapidly degraded allowing the cargo to get released into the lysosome where the degradation process starts (Nie et al., 2021). Hydrolases in the lysosome catabolize the cargo and the recycled components are released to ensure the reuse of the nutrients by the cell (Nie et al., 2021). In microautophagy, the selective targeting of cytosolic proteins is done through the Hsc70 to late endosomes using the same KFERQ motif as in the mechanism of CMA (Scrivo et al., 2018). This process is known as endosomal microautophagy (Scrivo et al., 2018).

CMA does not require the formation of membranous structures (Nie et al., 2021). In CMA, the cytosolic proteins to be degraded get translocated across the lysosomal membrane through a molecular chaperone complex in the cytosol that is associated with the lysosomal membrane (Dice, 2007; Mizushima and Levine, 2020). The translocation process starts with the recognition of a KFERQ-like pentapeptide sequence in the substrate protein by the heat shock protein of 70 kDa (Hsc70) and cochaperones (Dice, 2007; Mizushima and Komatsu, 2011). The substrate protein and chaperone complex interact with the lysosomal-associated membrane protein type 2A (LAMP2A) receptor located on the lysosomal membrane to be transported into the lysosome (Dice, 2007; Lynch-Day et al., 2012).

Autophagy is constitutively active and particularly important in post-mitotic cells such as neurons because they cannot use cell division to dilute protein aggregates or other cytosolic waste to prevent their accumulation (Nah et al., 2015). The special morphology of the neuron, large dendritic and axonal cytoplasm, makes it difficult for them to eliminate waste products in time to prevent cytotoxicity (Guo et al., 2018). In neuronal autophagy, key autophagy components are located at different places in the neuron (Guo et al., 2018). Autophagosomes formed in the axons travel long distances to reach the cell body where the lysosomes are located and fuse with the lysosomes to initiate the degradation of the autophagosome cargo (Guo et al., 2018). Consequently, autophagy alterations and impaired axonal transport make neurons highly susceptible to cell damage and death (Nah et al., 2015; Bar-Yosef et al., 2019). Neural autophagy is crucial as this mechanism plays an important role in axonal homeostasis, dendrite spine, and synapse formation (Wang et al., 2019; Valencia et al., 2021). Neuronal autophagy is also crucial to meet the energy demands and support synaptic plasticity because synapses require high energy and protein turnover (Bar-Yosef et al., 2019; Valencia et al., 2021).

Defective autophagy is closely associated with the pathophysiology of neurodegenerative disorders such as Parkinson’s disease (PD), Alzheimer’s disease (AD), amyotrophic lateral sclerosis (ALS), and Huntington’s disease (HD) and mood disorders such as depression (Jia and Le, 2015; Nah et al., 2015; Wang et al., 2019). Understanding the underlying molecular pathophysiology of neurological disorders and depression is crucial due to the prevalence of these disorders. The number of neurodegenerative disease cases is alarmingly increasing, and these disorders are becoming a major global cause of mortality and morbidity (Erkkinen et al., 2018; Santomauro et al., 2021). Estimations indicate that about 6.5 million people around the world are affected by AD (Alzheimer’s Association, 2022), about 1% of the global population is affected by PD, the prevalence of ALS accounts for 7.7 per 100,000 only in the USA (Mehta et al., 2023), the prevalence of HD is 10.6–13.7 cases per 100,000 in Western populations and 1 to 7 cases per million in Eastern populations (McColgan and Tabrizi, 2018) whereas depression affects about 10% of the global population (Alcocer-Gómez et al., 2017). Evidence suggests that the activation of autophagy could slow down or stop neurodegenerative changes (Jia and Le, 2015; Nah et al., 2015). For instance, recent studies have shown the potential therapeutic properties of autophagy inducers such as bromo-protopine, a protopine derivative that stimulates CMA and reduces the presence of pathological tau in AD models (Sreenivasmurthy et al., 2022) and klotho, a single-pass transmembrane protein in the brain, that induces autophagy leading to increased autophagy mediated clearance of amyloid-β in AD models (Fung et al., 2022). Moreover, research on the mechanism of autophagy and autophagy inducers contributes to the identification of autophagy targets with therapeutic relevance in neurodegenerative diseases such as KIF5B/kinesin-1, an autophagy-related target in AD (Selvarasu et al., 2022). In this context, cell-based methods to study autophagy in neurodegenerative diseases and mood disorders as well as to screen for autophagy modulators are research tools that play a pivotal role in understanding autophagy-mediated neuroprotection and therapeutics. Cell-based models are used to resemble relevant disease hallmarks and are powerful tools in drug discovery research to shed light on the identification of drug targets (Cetin et al., 2022). Therefore, the aims of this narrative review are (1) to compile information on the cell-based methods used in autophagy research, (2) to discuss their application, and (3) to create a catalog of traditional and novel *in vitro* methods applied in neurodegenerative diseases and depression. Our hypothesis was that traditional *in vitro* methods are still being used as they are designed to explore relevant and well-known molecular key players in autophagy research of neurodegenerative disorders and depression. Also, we hypothesized that new methods applied to autophagy research have emerged thanks to technological advances. Therefore, the analysis and compilation of both traditional and novel *in vitro* methods can guide autophagy researchers in the field of neurological disorders and depression to adapt their experimental designs for future autophagy research. The catalog of *in vitro* models applied to autophagy research of neurodegenerative disorders and depression include a repertoire of traditional and novel approaches and techniques. These *in vitro* tools can be applied to explore pathophysiological mechanisms at a molecular level and to screen for potential therapeutic agents and their mechanism of action.

## 2. Methodological considerations of this review

**Figure 1:**
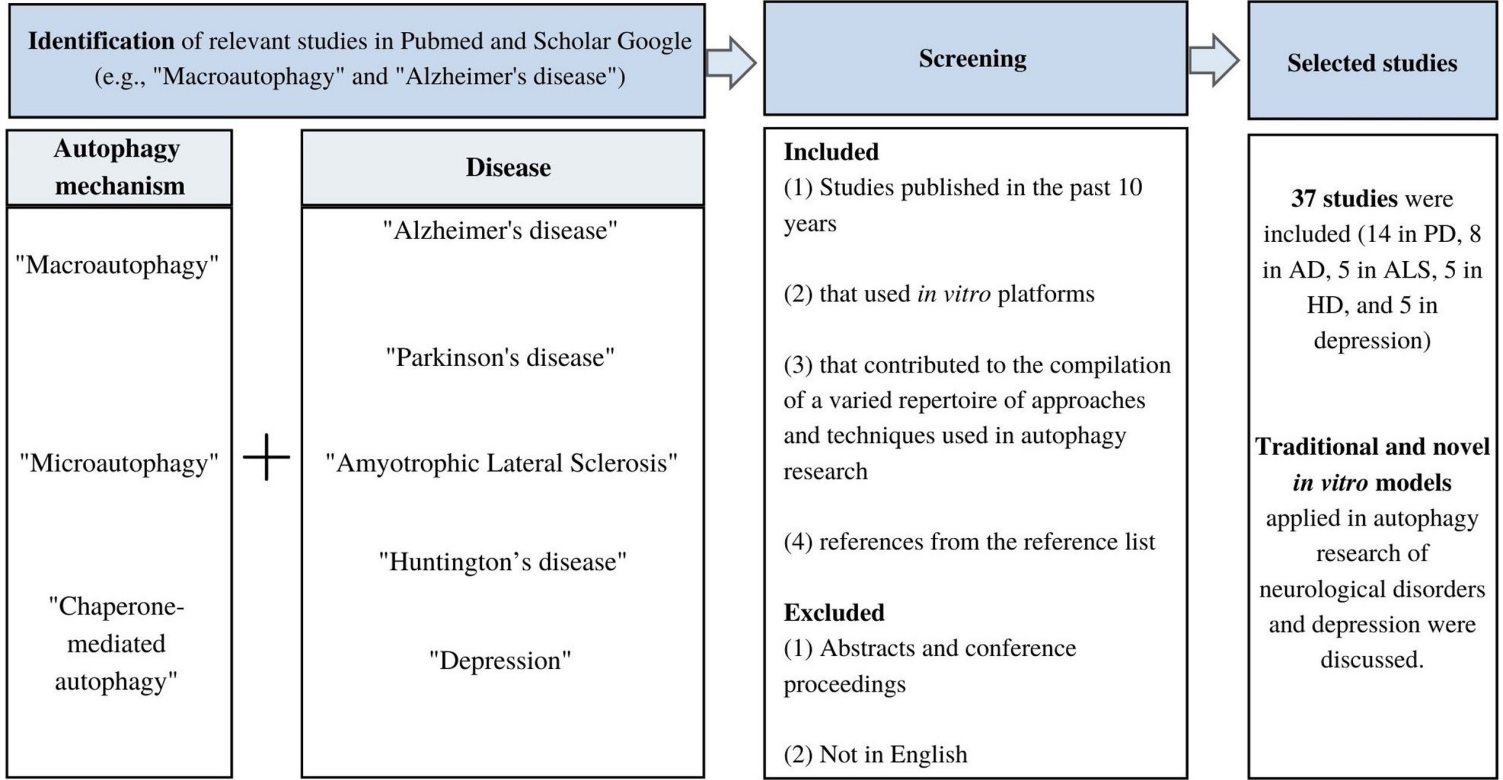
Flowchart of study screening and selection.

A narrative review was conducted to qualitatively summarize the current *in vitro* methods used in autophagy research in neurodegenerative diseases and mood disorders. Pubmed and Google Scholar were used to retrieve relevant *in vitro* studies on autophagy mechanisms in neurological diseases and depression using a combination of search terms per mechanism and disease (Figure 1). The terms “macroautophagy” and “Alzheimer’s disease” were used to retrieve studies on macroautophagy in AD. Since this review is a narrative review rather than a systematic review, no record of the total number of articles retrieved or excluded was kept. Instead, a qualitative screening of articles took place considering the following: (1) studies published in the past 10 years (2) that used *in vitro* platforms, and (3) allowed the compilation of a varied repertoire of approaches and techniques used in autophagy research in neurodegenerative disorders and depression. Studies in English were included and abstracts and conference proceedings were excluded. Studies published more than 10 years ago were only considered when the *in vitro* methods are still used and add-up to the variety of the list of methods presented. Furthermore, the reference list of relevant studies was revised to identify articles that could be included. This is the first review article compiling relevant information on the application of *in vitro* methods in autophagy research focused on neurodegenerative diseases and mood disorders. The information in the review is presented starting with a discussion of the role of autophagy in neurodegenerative disorders, including PD, AD, ALS, and HD, and mood disorders such as depression to establish the key autophagy molecular players in each disorder. Next, the cell-based methods used to investigate relevant autophagy-related mechanisms and to screen for autophagy mediators are discussed. The effect of therapeutic strategies on autophagy was not considered in the present review. Instead, emphasis was put on cell-based platforms and their application in the study of autophagy. A total of 37 studies were included (14 in PD, 8 in AD, 5 in ALS, 5 in HD, and 5 in depression).

## 3. Parkinson’s disease (PD)

First reported by the British doctor James Parkinson in 1817, PD is nowadays the second most common neurodegenerative disorder after AD that affects the motor system (Guo et al., 2018; Hou et al., 2020). This chronic movement disorder gradually develops over time and is prevalent in 1% of the elderly population over 60 years of age and in 5–6% of the population over 85 years old (Hou et al., 2020). PD is characterized by resting tremors, muscle rigidity, and bradykinesia as a result of selective loss of dopamine neurons in the substantia nigra pars compacta and the accumulation of mutant proteins in inclusions of Lewy bodies (Winslow et al., 2010; Guo et al., 2018; Bar-Yosef et al., 2019). The majority of PD cases are sporadic in which the etiology is unknown while 5% of the cases are hereditary and have been associated with mutations (Nah et al., 2015). Mutations in the genes encoding the proteins α-synuclein, leucine-rich repeat kinase 2 (LRRK2), vacuolar protein sorting 35 (VSPS35), the chaperone protein REM-8, and the gene CHCHD2 encoding a mitochondrial protein have been found in cases of autosomal dominant PD (Moloudizargari et al., 2017). Mutations in genes encoding proteins such as the ubiquitin-ligase parkin, PTEN-induced kinase 1 (PINK1), and the mitochondrial protein DJ-1 involved in the regulation of oxidative stress have been reported in autosomal recessive cases of PD (Moloudizargari et al., 2017; Bar-Yosef et al., 2019; Meng et al., 2019). Other rare mutations include the regulators of autophagy ATP13A2, the lysosomal hydrolase glucocerebrosidase β acid (GBA), and the lysosomal integral membrane protein-2 (LIMP-2) (Moloudizargari et al., 2017). In PD, mutant proteins act at different stages of autophagy through different mechanisms. Misfolded and aggregated mutant α-synuclein and polyubiquitinated proteins are the main component of Lewy bodies, one of the key pathological hallmarks in PD suggesting that insufficient protein clearance leads to aberrant protein accumulation (Guo et al., 2018; Meng et al., 2019). Post-mortem samples from PD patients show abnormal accumulation of autophagosomes in neurons which indicates that dysfunctional autophagy may play a role in PD (Guo et al., 2018; Bar-Yosef et al., 2019). Mitochondrial dysfunction, and oxidative stress, all of which are closely associated with autophagy, also contribute to the pathogenesis of PD (Lynch-Day et al., 2012).

### 3.1. Macroautophagy in PD

Dysfunctional macroautophagy is implicated in PD (Nah et al., 2015). α-synuclein has been considered one of the crucial key players as it is the main component of Lewy bodies observed in PD brains (Lu et al., 2020). This protein is prone to form highly toxic aggregates, especially the mutant forms, and the formation of Lewy bodies (Lu et al., 2020). Mutant forms of α-synuclein can affect both the autophagosome-mediated and CMA degradation pathways leading to different levels of toxicity in the cell (Lynch-Day et al., 2012). Degradation of all forms of α-synuclein can be conducted through the macroautophagy pathway (Guo et al., 2018). At the elongation phase of macroautophagy, α-synuclein, a pre-synaptic regulator of dopamine neurotransmission, inhibits autophagy as its accumulation causes mislocalization of the autophagy protein Atg9, which affects autophagosome formation (Kim and Guan, 2015; Bar-Yosef et al., 2019). Although the presence of mutant α-synuclein suppresses autophagy contributing to its accumulation and cytotoxicity (Meng et al., 2019), the accumulation of α-synuclein can also be triggered by other mechanisms. For instance, changes in post-translational modifications, such as higher lysine acetylation levels in histones (e.g., SIRT2 that deacetylates α-synuclein) also contribute to the accumulation of α-synuclein in dopaminergic neurons of PD patients (Wang R. et al., 2020). Therefore, mutations should be regarded as part of the array of triggers associated with the accumulation of α-synuclein. Other mutations associated with PD are VPS35, a protein involved in the trafficking of proteins in the cytoplasm, and LRRK2 proteins are observed in autosomal dominant PD cases (Moloudizargari et al., 2017; Meng et al., 2019). Mutant VPS35 affects autophagosome formation through mislocalization of the autophagy protein Atg9 (Guo et al., 2018; Meng et al., 2019). Autophagosome aggregation and recruitment of PI3KIII complex and Rubicon, a negative regulator of autophagy, have been observed in the presence of mutant LRRK2 causing deficits in autophagy, shortening of dendrites, and inhibition of phagosome maturation leading to autophagosome aggregation (Guo et al., 2018; Meng et al., 2019; Nakamura et al., 2019). At the lysosome level, alterations in the lysosomal hydrolase GBA affect the autophagosome-lysosome mechanism leading to the accumulation of α-synuclein (Meng et al., 2019). Lysosomal ATPases play an essential role in maintaining the optimal conditions in the lysosome (e.g., lysosomal pH) for protease activity (Meng et al., 2019). Mutant ATP13A2 causes down regulation of the degradation processes taking place inside the lysosomes leading to the accumulation of α-synuclein in dopaminergic neurons and mutant LIMP-2 is involved in dysfunctional autophagosome and lysosome function (Meng et al., 2019). Parkin and PINK1 are proteins involved in the selective degradation of damaged mitochondria by mitophagy (Guo et al., 2018). Mutations in both parkin and PINK1 affect the recruitment of autophagy components leading to reduced mitophagy (Bar-Yosef et al., 2019; Broadway et al., 2022). Impaired mitophagy caused by mutant parkin and PINK1 enhances sensitivity to oxidative stress due to the accumulation of dysfunctional mitochondria and excessive production of reactive oxygen species which has been associated with PD (Lynch-Day et al., 2012; Meng et al., 2019). TMEM175, a component of the lysosome proteome responsible for the regulation of lysosomal pH and function, plays a role in mitophagy by influencing respiration and regulation of energy homeostasis in the mitochondria (Meng et al., 2019).

### 3.2. Microautophagy in PD

Microautophagy has also been involved in the pathophysiology of PD, and this autophagy process involves intricate coordination of multiple mediators (Perrett et al., 2015) Structures such as the late endosome and multivesicular bodies are responsible for the selective uptake of proteins in microautophagy (Krause and Cuervo, 2021). The Hsc70 protein binds to the KFERQ-like motif present in cytosolic proteins, and the Hsc70-labeled proteins get engulfed into the multivesicular body compartment for lysosomal degradation (Krause and Cuervo, 2021). Other key players participate in microautophagy, including proteins such as Rab5 and Rab7 that regulate endosomal maturation, VPS35 and REM-8 that participate in actin polymerization, and SNARE proteins that contribute to the fusion of the multivesicular bodies with the lysosome (Perrett et al., 2015). Genetic mutations found in α-synuclein as well as mutations in microautophagy regulators (e.g., VPS3) suggest aberrant microautophagy in PD (Perrett et al., 2015; Krause and Cuervo, 2021). Other molecular players and signaling pathways also interfere with the microautophagy degradation process. For instance, the neutral sphingomyelinase 2 (nSMase2)- and ESCRT-mediated signaling pathways are possibly involved in regulating the uptake of α-synuclein by endosomal multivesicular bodies (Sackmann et al., 2019). The relationship between microautophagy and the above-mentioned pathways was evident when the inhibition of nSMase2 led to a significant reduction in the cell-to-cell transfer of oligomeric α-synuclein between neuron-like cells and decreased the aggregation of α-synuclein (Sahu et al., 2011; Sackmann et al., 2019). Moreover, α-synuclein with Lys-63 polyubiquitin chains could be taken up by late endosomes in an ESCRT-dependent manner and subsequently degraded by the lysosomes (Sackmann et al., 2019). Another kay player that participates in the microautophagy-mediated degradation of α-synuclein is a neural precursor cell expressed developmentally down-regulated protein 4, known as Nedd4 (Sackmann et al., 2019). Nedd4 regulates the ubiquitination of Lys-63 residues present in α-synuclein and promotes α-synuclein internalization in SH-SY5Y cells (Sugeno et al., 2014; Sackmann et al., 2019). The Needd4-mediated ubiquitination mechanism is in turn regulated by CHMP2B (Sugeno et al., 2014; Sackmann et al., 2019).

### 3.3. Chaperone-mediated autophagy in PD

Alterations in the CMA mechanism have also been implicated in the pathophysiology of PD (Nah et al., 2015). The presence of mutant α-synuclein can affect both macroautophagy and CMA (Bar-Yosef et al., 2019). In normal conditions, α-synuclein is translocated into the lysosomes to undergo degradation by the CMA machinery (Bar-Yosef et al., 2019). However, the mutant form of α-synuclein interrupts the CMA process by binding to LAMP2A and thus blocking it and affecting the protein translocation into the lysosome for degradation (Nah et al., 2015; Bar-Yosef et al., 2019). A53T mutant α-synuclein and A30P mutant α-synuclein can strongly bind to the LAMP2A receptor preventing binding the degradation of other targets through the CMA pathway (Lynch-Day et al., 2012; Lu et al., 2020). Furthermore, the mutant form of the LAMP2A receptor disrupts the degradation pathway mediated by CMA leading to the accumulation of toxic aggregates in the cytoplasm as observed in PD post-mortem tissues (Lynch-Day et al., 2012). Blockade of the LAMP2A receptor leads to the upregulation of autophagy as a compensatory response that may trigger cell death and the accumulation of toxic components in the cell (Das et al., 2012; Bar-Yosef et al., 2019). For instance, LRRK2 is degraded by CMA but not the mutant form, thus blockade of the CMA degradation pathway will impair the normal process of degradation of LRRK2 leading to its accumulation in the cell (Nah et al., 2015). DJ1, which is a mitochondrial protein involved in the regulation of oxidative stress, has also been associated with PD (Meng et al., 2019). Overexpression of DJ1 in astrocytes can have a positive effect on CMA suggesting that the presence of the mutant DJ1 could affect CMA (Meng et al., 2019). Inhibition of the CMA pathway promotes the buildup of toxic compounds in the neuron through the formation of aggregates and prevents the protective activity of transcription factors crucial in neuronal survival such as the protein myocyte enhancer factor 2D (MEF2D) (Lynch-Day et al., 2012; Lu et al., 2020). MEF2D activity is regulated by CMA-dependent degradation. When the CMA pathway is inhibited, there is an accumulation of inactive MEF2D which cannot longer bind to the DNA to assist in the transcription process (Lynch-Day et al., 2012).

### 3.4. Autophagy *in vitro* methods in PD research

Several *in vitro* platforms and analytical techniques have been used to understand the contribution of autophagy-mediated degradation to the pathogenesis of PD. Table 1 summarizes studies that used PD models on different mechanisms of autophagy and molecular targets, and a brief description of the application and readout of the cell-based methods used.

**Table 1:** *In vitro* autophagy methods in Parkinson’s disease research.

Autophagy mechanism	Autophagy stage	Molecular target	Cell line	Application (A), description (D), and readout (R)	References
MA	Autophagy induction and autophagic flux	Mutant α-synuclein, transcription factor EB (TFEB), and MA biomarkers	PC12 and HeLa cells	**A**: Evaluation of a mutant form of α-synuclein and identification of biomarkers at different stages of MA using the autophagy activator rapamycin and AMPK inhibitor compound C. **D**: The generation of mutant α-synuclein was induced by treatment with doxycycline. Phosphorylated and non-phosphorylated mutant α-synuclein were measured to assess its autophagy-mediated clearance. Autophagy induction (Ulk1, AMPK, mTOR), TFBE activation, autophagic flux (LC3I and LC3II), and lysosomal biogenesis (LAMP1) were measured. **R**: (1) Protein expression of the phosphorylated and non-phosphorylated form of α-synuclein by Western blot. (2) Nuclear translocation of TFEB by immunostaining and Western blot of flag-TFEB. (3) Protein expression of LC3I and L3CII by Western blot. (4) Protein activation by measuring Ulk1 and p-Ulk1, AMPK, p-AMPK, mTOR, and p-mTOR using Western blot.	Xu et al. (2022)
MA	Autophagy induction, autophagic flux, and autophagosome maturation	MA biomarkers	Ventral midbrain dopaminergic neurons (mDANs) derived from human induced pluripotent stem cells (hiPSC)	**A**: Monitoring MA markers specific to different stages of the autophagy process using cells treated with an autophagy activator (AZD8055) and an inhibitor of autophagosome-lysosome fusion (bafilomycin A1, BafA1). **D**: Upon autophagy activation by AZD8055, MA markers were measured. An autolysosome-fusion inhibitor was used to assess autolysosome formation. **R:** (1) Gene expression of MA markers (beclin 1, LC3, p62, Optn, Ambra1) by qRT-PCR. (2) Protein expression by immunostaining targeting LC3 and WIPI2, Western blot (LC3 and p62), and detection of green fluorescent protein-tagged LC3 and Atg5 by time-lapse wide-field microscopy. (3) Quantitation of autophagic vacuoles and autophagic flux using the CYTO-ID autophagy kit that labels accumulated autophagic vacuoles. (4) Analysis of autolysosomal vacuoles by ultrastructural analysis using electron microscopy.	Stathakos et al. (2021)
MA	Autophagic flux and autolysosome maturation	Sirtuin proteins which are involved in cell survival mechanisms.	Lund human mesencephalic (LUHMES) cells	**A**: Degradation of several sirtuin proteins in dopaminergic neuronal cells using different PD models. **D**: LUHMES cells were subjected to 1-methyl-4-phenylpyridinium, rotenone, and paraquat, which inhibit autophagy, and the autophagy-mediated degradation of sirtuin proteins in the presence or absence of the autophagosome-lysosome fusion inhibitor BafA1 was monitored. **R**: (1) Protein expression of sirtuin proteins and the MA mediator LC3B by Western blot and immunostaining.	Baeken et al. (2021)
MA	Expansion	α-synuclein and Atg5	Primary cells	**A**: Assessment of the expression of the autophagy marker Atg5 by treating the cells with an autophagy inhibitor (3-ethyladenine, 3-MA) and oligomerized α-synuclein. **D**: Cells were treated with different concentrations of oligomeric α-synuclein and the levels of Atg5 were monitored. **R**: Protein expression by Western blot analysis of Atg5.	Wang X. et al. (2020)
MA	Autophagy induction and autophagic flux	α-synuclein, autophagy induction, and autophagosome formation markers	PC12 cells	**A**: Identification of induction and autophagosome formation using the markers beclin 1, p62, mTOR, and phospho-mTOR, and LC3I/LC3II after treatment with an autophagy inhibitor (rotenone) and a neuroprotective agent (icariin) to assess autophagy induction and autophagic flux. **D**: Cells were pretreated with a neuroprotective agent and then challenged with an autophagy inhibitor. **R**: (1) Protein expression by Western blot analysis targeting α-synuclein and autophagy markers. (2) Cell viability by lactate dehydrogenase assay.	Zeng et al. (2019)
MA	Autophagy induction and autophagic flux	MA biomarkers and autophagosome morphology	PC12 cells	**A**: Detection of MA induction and autophagic flux upon treatment with a neurotoxin was used as a PD model. An autophagy activator (rapamycin) and an autophagy inhibitor (3-MA) were also used. **D**: Cells were treated with a neurotoxin as a PD model. Autophagy markers were monitored upon treatment with an autophagy activator and inhibitor. **R**: (1) Protein expression by Western blot analysis of beclin 1, LC3, and Atg12. (2) Gene expression by mRNA analysis of beclin 1, LC3, and Atg12 by qRT-PCR. (3) Autophagosome formation by identification of double-membrane enclosed autophagosome by transmission electron microscopy.	Liu et al. (2019)
MA	Autophagosome maturation and autophagic flux	Park9 (ATP13A2), an ATPase found on the lysosomal membrane and Atg7	SH-SY5Y and primary neuronal cells	**A**: Study of the role of Park9 and Atg7 in autophagic flux and autophagosome maturation in SH-SY5Y cells and primary neurons. **D**: Small interfering RNA was used to knock down Park9 and Atg7 in both types of cells. A gene-reported approach was used to tag LC3 with a green and red fluorescent protein. Dual green and red positive cells indicated early autophagosomes and red fluorescence indicated autophagosome maturation. **R**: (1) Autophagic flux was assessed by Western blot targeting LC3II and LC3I. (2) Autophagy maturation was assessed in green fluorescent and red fluorescent protein-tagged LC3 cells.	Gusdon et al. (2012)
MA and CMA	MA: Autophagy induction and autophagic flux CMA: Substrate degradation	α-synuclein, MA and CMA biomarkers, and CMA substrate MEF2D	SH-SY5Y cells	**A**: Monitoring the degradation of monomeric and oligomeric forms of α-synuclein and a CMA substrate using autophagy inhibitors (rotenone and ammonium chloride). **D**: Cells were treated with autophagy inhibitors to assess the degradation of different forms of α-synuclein. MA induction and autophagosome formation were monitored to evaluate α-synuclein degradation. Cells were treated with the CMA substrate MEF2D to assess its degradation. **R**: (1) Gene and (2) protein expression of α-synuclein and autophagy markers of induction and autophagosome formation (beclin1, LC3I, LC3II, p62) and CMA markers (LAMP2A, Hsc70) were assessed using qRT-PCR and Western blot, respectively. (3) Substrate degradation by measuring the extracellular concentration of α-synuclein was assessed by Dot blot.	Sala et al. (2021)
MA and CMA	MA: Autophagy induction and autophagic flux CMA: Cargo translocation to the lysosome through LAMP2A	LRRK2, LAMP2A, α-synuclein, and MA biomarkers	Patient-specific induced pluripotent stem cell (iPSC)-derived astrocytes and ventral midbrain dopaminergic neurons (vmDAns) from PD patients with LRRK2 mutation and genetically engineered iPSC cell lines using CRISPR/Cas9 technology to tag α-synuclein	**A**: Monitoring CMA-mediated degradation of α-synuclein and MA biomarkers in cells derived from PD patients with a mutation in the LRRK2 gene and healthy volunteers. Also, the role of mutant LRRK2 and the downregulation of LAMP2A was assessed by cell engineering. **D**: α-synuclein was detected in both cells derived from patients and healthy subjects and engineered cells to assess CMA-mediated degradation of α-synuclein and flagged α-synuclein. Also, LAMP2A was knocked down in astrocytes. MA biomarkers to assess induction and autophagosome formation were measured. **R**: (1) CMA-mediated degradation of α-synuclein by measuring the accumulation of α-synuclein and transfer of α-synuclein using flagged α-synuclein assessed by immunostaining. (2) Protein expression by immunostaining and Western blot targeting LAMP2A, α-synuclein, LC3II, LC3I, and p62.	di Domenico et al. (2019)
MA and CMA	MA: Autophagic flux CMA: Cargo translocation to the lysosome through LAMP2A	α-synuclein and MA biomarkers	Human adipose neural crest stem cells (haNCSCs) from subjects with PD and GBA1 mutation, a gene associated with increased α-synuclein	**A**: Monitoring the MA and CMA degradation of α-synuclein in cells derived from PD patients with GBA1 mutation to study α-synuclein degradation mediated by MA and CMA. **D**: An autophagy inducer (ambroxol) was used to assess autophagic flux and CMA. OR: (1) Protein expression of autophagy and autophagic flux markers (LC3II, LC3I, and p62) and CMA autophagy (LAMP2A and LAMP1) by Western blot. (2) Substrate degradation (α-synuclein) by immunostaining.	Yang et al. (2017)
MA and CMA	CMA: Recognition of motif MA: Cargo recognition	Wild type (WT) and mutant α-synuclein LAMP2A	PC12 andSH-SY5Y cells Primary neuronal cells	**A1**: Identification of the CMA pathway as a major route of degradation for WT α-synuclein. WT and mutant α-synuclein turnover by activating autophagy with doxycycline in cells overexpressing of α-synuclein and mutant α-synuclein and well as downregulated LAMP2A receptor to decrease CMA-mediated degradation. **A2**: Identification of the contribution of MA-dependent degradation of wild type α-synuclein. Proteasome (epoxomicin) and MA (3-MA) inhibitors were used. **D**: Overexpression of α-synuclein and expression of a mutant form of α-synuclein was induced in PC12 and SH-SY5Y cells. LAMP2A expression in PC12 was downregulated in PC12 cells to block the CMA pathway. Primary cultures were used as reference. **R**: (1) Half-life of WT (endogenous and overexpressed) and mutant α-synuclein in all cell types using radiolabeling and subsequent immunoprecipitation against α-synuclein. (2) Protein expression by Western blot analysis targeting α-synuclein, LAMP2A, and ubiquitin and by immunocytochemistry targeting α-synuclein. (3) Gene expression of α-synuclein by RT-PCR.	Vogiatzi et al. (2008)
MA and CMA	MA: Autophagic flux CMA: Cargo translocation to the lysosome through LAMP2A	α-synuclein, Atg5, and LAMP2A	Rat oligodendroglial OLN-93, OLN-AS7 (overexpressing human α-synuclein) and OLN-p25α (overexpressing human tubulin polymerization promoting protein, TPPP/p25α) cell lines	**A**: Evaluation of MA and CMA degradation of α-synuclein in engineered cells overexpressing human α-synuclein and human TPPP as well as cells with downregulation of LAMP2A and Atg5. **D**: Cells overexpressing α-synuclein and TPPP were treated with the inhibitor 3-MA and CMA lysosomal inhibitor (ammonium chloride) and α-synuclein degradation was monitored. Downregulation of MA and CMA markers was conducted while monitoring α-synuclein degradation. **R**: (1) Protein expression of MA markers (LC3II, LC3I, p62), (2) Gene expression of MA (Atg5) and CMA (LAMP2A). (3) Accumulation of α-synuclein by immunostaining and Western blot.	Mavroeidi et al. (2022)
CMA	CMA: Cargo translocation to the lysosome through LAMP2A	α-synuclein, LAMP2A, and Hsc70	SH-SY5Y and SKMEL-28 cells	**A**: Role of CMA in the degradation of α-synuclein in normal cells and cells overexpressing wild-type α-synuclein. Also, cells were transfected with eight miRNAs identified in PD brains that regulate the expression of LAMP2A and Hsc70. **D**: Cells were challenged with stressors (rotenone and paraquat). The expression of LAMP2A, Hsc70, and α-synuclein was assessed. Regulation of LAMP2A and Hsc70 was assessed using a luciferase reporter assay transfected with miRNAs derived from PD brains. **R**: (1) Protein and (2) gene expression of LAMP2A, Hsc70, and α-synuclein by Western blot and qRT-PCR. (2) Gene expression of LAMP2A and Hsc70 by luciferase reporter assay.	Alvarez-Erviti et al. (2013)
MiA	Endosomal multivesicular bodies	α-synuclein	SH-SY5Y cells	**A**: Identification of inhibition of neutral sphingomyelinase 2 (nSMase2) expression, a hydrolyze that acts on the sphingolipid sphingomyelin to produce ceramide that forms extracellular vesicles. Increased levels of ceramide have been found in PD patients. **D**: Inhibition of nSMase2 prevents oligomeric α-synuclein aggregation in SH-SY5Y cells. **R**: (1) Knockdown of nSMase2 using siRNA transfection and CRISPR/Cas9 technique. (2) Protein expression by Western blot analysis targeting nSMase2 and α-synuclein. (3) Assessment of nSMase2 activity by enzyme activity assay.	Sackmann et al. (2019)

MA, macroautophagy; MiA, microautophagy; CMA, chaperone-mediated autophagy.

Macroautophagy and CMA are most commonly studied in PD with only a few studies focusing on microautophagy. Studies on macroautophagy are mainly concerned with autophagy induction, expansion, autophagosome maturation, and autophagic flux, while CMA studies focus on the cargo recognition, translocation, and substrate degradation steps, and studies on microautophagy are limited to the formation of endosomal multivesicular bodies (Table 1). Monitoring the accumulation and degradation of α-synuclein as well as the use of autophagy inhibitors and activators, cell engineering methods, and the use of cells from PD patients are common approaches in autophagy research of PD using *in vitro* systems.

Measuring the accumulation of different forms of α-synuclein is crucial in *in vitro* autophagy research of PD (Guzzo et al., 2022). In this line, either monitoring of α-synuclein accumulation (Xu et al., 2022) or exposing the cells to α-synuclein (e.g., oligomeric α-synuclein) (Wang X. et al., 2020) to induce a pathological state are approaches used in PD research. Protein expression analysis by Western blot is commonly used to measure different forms of α-synuclein such as monomeric (Alvarez-Erviti et al., 2013; di Domenico et al., 2019; Sackmann et al., 2019; Zeng et al., 2019; Sala et al., 2021; Mavroeidi et al., 2022), oligomeric (Sala et al., 2021), and phosphorylated α-synuclein (Xu et al., 2022). About 90% of the α-synuclein aggregates found in Lewy Bodies of brain tissue from PD patients are phosphorylated at serine 129 (Ser129-phosphorylated α-synuclein) (Sasaki et al., 2015). The analysis of Ser129-phosphorylated α-synuclein is crucial as high concentrations of phosphorylated α-synuclein is an early event that promotes α-synuclein aggregation (Sasaki et al., 2015). Therefore, measurement of monomeric α-synuclein that is available for phosphorylation and subsequent degradation, measurement of the phosphorylated form, which accelerates the aggregation process, or measurement of the oligomeric form which represents a late state of α-synuclein aggregation should be considered *a priori* in the design of an *in vitro* experiment to assess α-synuclein degradation. Other methods to monitor α-synuclein accumulation and degradation include gene expression analysis by qRT-PCR (Alvarez-Erviti et al., 2013), immunostaining (Yang et al., 2017; Mavroeidi et al., 2022), immunohistochemistry (Vogiatzi et al., 2008), radiolabeling and subsequent immunoprecipitation (Vogiatzi et al., 2008), and dot blot (Sala et al., 2021). However, those methods are mainly used to detect monomeric and non-phosphorylated α-synuclein. In terms of the evaluation of specific stages or molecular players of the autophagy process, in addition to the traditional methods mentioned above, more sophisticated methods include the CYTO-ID autophagy kit that labels accumulated autophagic vacuoles for the quantitation of autophagic vacuoles and autophagic flux (Stathakos et al., 2021), time-lapse wide-field microscopy for the detection of green fluorescent protein-tagged LC3 and Atg5 (Stathakos et al., 2021), ultrastructural analysis using electron microscopy for the analysis of autolysosomal vacuoles (Stathakos et al., 2021), transmission electron microscopy to assess autophagosome formation by identification of double-membrane enclosed autophagosomes (Liu et al., 2019), and cell engineering to generate green fluorescent and red fluorescent protein-tagged LC3 cells to evaluate autophagy maturation (Gusdon et al., 2012).

The activation and inhibition of autophagy mechanisms are used to understand the regulation and contribution of autophagy and to identify potential drug targets (Yang et al., 2013). Activators and inhibitors act at different stages of the autophagy pathway. Activators of the macroautophagy pathway include rapamycin (Liu et al., 2019; Xu et al., 2022), AZD8055 (Stathakos et al., 2021), and ambroxol (Yang et al., 2017). Rapamycin is a drug that inhibits the mTOR signaling pathway, thus leading to the activation of the macroautophagy leading to a reduced accumulation of α-synuclein (Moors et al., 2017). The rapamycin-mediated activation of macroautophagy starts with the formation of a complex with the FK506-binding protein that subsequently binds to and inhibits the activity of mTOR kinase (Ghosh and Pattison, 2018). Rapamycin also acts on other autophagy regulators. For instance, it activates Atg1 and improves the binding affinity of Atg1 to Atg13 and Atg17 (Ghosh and Pattison, 2018). Another activator that acts at the induction stage of macroautophagy is the compound AZD8055, a potent mTOR inhibitor (Hu et al., 2014; Stathakos et al., 2021). Autophagy activation can also be induced at other stages. For instance, ambroxol acts at the lysosomal level (Yang et al., 2017; Magalhaes et al., 2018). This compound is an expectorant drug that promotes the translocation of glucocerebrosidase, an enzyme that breaks down glucocerebroside into glucose and ceramide in the lysosome, to the lysosome improving lysosomal enzymatic activity and reducing oxidative stress (McNeill et al., 2014; Moors et al., 2017). Concerning autophagy inhibitors, the neurotoxic agent rotenone is commonly used in PD cell models (Alvarez-Erviti et al., 2013; Zeng et al., 2019; Baeken et al., 2021; Sala et al., 2021). Rotenone, a herbicide, and pesticide is a neurotoxin found in tropical plants that can cross the blood–brain barrier (Salari and Bagheri, 2019). It damages dopaminergic neurons and causes aggregation of α-synuclein and Lewy body inclusions (Salari and Bagheri, 2019). Rotenone promotes cell death through the induction of mitochondrial dysfunction, increases oxidative stress, aggregation of α-synuclein, post-translational modifications, and accumulation of autophagy vacuoles leading to neuronal death (Mader et al., 2012; Ioghen et al., 2023). Paraquat (Alvarez-Erviti et al., 2013; Baeken et al., 2021) is a herbicide that affects the mitochondria causing increased oxidative stress that leads to cellular damage and eventually death of dopaminergic neurons (Salari and Bagheri, 2019). Similar to ratonone, paraquat causes the accumulation of autophagy vacuoles (Lynch-Day et al., 2012). 3-methyladenine (3-MA) is another compound used to inhibit autophagy (Vogiatzi et al., 2008; Liu et al., 2019; Wang X. et al., 2020; Mavroeidi et al., 2022). 3-MA, a PI3K inhibitor, acts at the induction phase of macroautophagy by blocking the mTOR-mediated activation pathway (Albanese et al., 2019). At the autophagosome level, 3-MA inhibits the formation of autophagosomes (Ghosh and Pattison, 2018). The compound 1-methyl-4-phenylpyridinium (MPP+), a metabolite of 1-methyl-4-phenyl-1,2,3,6-tetrahydropyridine (MPTP), a gold standard inducer of PD in research settings, that selectively damages dopaminergic neurons, exhibits high affinity to the dopamine transporter leading to its accumulation inside the cells (Salari and Bagheri, 2019; Ioghen et al., 2023). MPP+ disrupts the mitochondrial electron transport chain increasing oxidative stress and causing cell damage and neuronal death (Ioghen et al., 2023). The antibiotic bafilomycin A1 (BafA1) is used as an autophagy inhibitor (Baeken et al., 2021; Stathakos et al., 2021). BafA1 acts at the lysosome level through the inhibition of the vacuolar H + ATPase that regulates the pH inside the lysosome (Redmann et al., 2016). Therefore, BafA1 disrupts the autophagy pathway by preventing the acidification of the endosomes and lysosomes, altering autophagosome maturation (Redmann et al., 2016). Acidification is crucial for the proper fusion of the autophagosome and lysosome which will ultimately lead to the degradation of the sequestered cargo in the autolysosome (Yamamoto et al., 1998). Ammonium chloride is used as an autophagy inhibitor that acts at the lysosome level similarly to BafA1 (Sala et al., 2021; Mavroeidi et al., 2022). Ammonium chloride inhibits the fusion of the autophagosome with the lysosome and therefore interrupts cargo degradation (Ghosh and Pattison, 2018).

Another approach applied to study autophagy is cell engineering to generate cell profiles relevant to PD. Cells of rat origin can be modified to overexpress human α-synuclein and human tubulin polymerization promoting protein, TPPP/p25α (Mavroeidi et al., 2022). More recently, cell reprogramming to generate induced pluripotent stem cells (iPSC) from PD patients can be used to obtain disease-specific iPSC to mimic cellular and molecular mechanisms involved in the pathophysiology of PD (Torrent et al., 2015; Yang et al., 2017; di Domenico et al., 2019). Astrocytes and ventral midbrain dopaminergic neurons can be obtained using patient-specific iPSC (di Domenico et al., 2019). In this model, cells from PD patients with LRRK2 mutation can be engineered using CRISPR/Cas9 technology (di Domenico et al., 2019). Subsequently, the CMA-mediated degradation of α-synuclein and macroautophagy markers can be monitored to assess autophagy-mediated degradation and the contribution of different autophagy-mediated mechanisms (di Domenico et al., 2019). Another example of a cell reprograming method in a PD model is the use of human adipose neural crest stem cells (haNCSCs) from subjects with PD and GBA1 mutation which is a gene linked to increased α-synuclein production (Yang et al., 2017). Cell reprograming offers the opportunity to address questions specific to relevant molecular key players, genetic contributors, and patient-specific profiles in PD (Torrent et al., 2015).

## 4. Alzheimer’s disease

AD is the most prevalent neurodegenerative disease (Li et al., 2017). Only in the USA, about 6.5 million people aged 65 years or above are currently living with AD and estimates indicate that the number of cases could increase to 13.8 million by 2060 (Alzheimer’s Association, 2022). AD is the most common type of dementia characterized by progressive loss of memory and cognitive function (Li et al., 2017). As the disease progresses, other symptoms such as confusion, irritability, aggression, mood swings, and reading and writing difficulties manifest (Nah et al., 2015). The hallmarks of AD include the presence of two abnormal structures, namely senile plaques consisting of extracellular amyloid-β (AB) deposits distributed along axons and dendrites and intra-neural neurofibrillary tangles aggregated into filaments inside the cells (Li et al., 2017; Guo et al., 2018; Bar-Yosef et al., 2019). AB plaques form as a result of aggregation of cleaved products of the amyloid precursor protein (APP) while neurofibrillary tangles are composed of hyperphosphorylated tau protein which is prone to form aggregates (Guo et al., 2018). Both aberrant structures lead to the progressive loss of neurons followed by the disruption of synapses leading to the clinical manifestations observed in AD (Li et al., 2017). Although the etiology of AD is now clear, recent evidence points out dysfunctional autophagy that fails to clear misfolded, hyperphosphorylated proteins, or protein aggregates preceding the formation of AB plaques or neurofibrillary tangles (Li et al., 2017). Some components involved in the APP catalytic pathway are key contributors to the formation of AB plaques and are associated with dysfunctional autophagy. An important molecular player in AD is presenilin 1 (PS1), a transmembrane protein ubiquitously expressed, which participates in the cleavage of APP as part of the APP-cleaving γ-secretase complex (Guo et al., 2018; Bar-Yosef et al., 2019). In normal conditions, APP is cleaved by β-secretase to generate the β-C-terminal fragment that is later cleaved by PS1 to produce Aβ (Guo et al., 2018). In AD, a mutant form of PS1 has been suggested to interfere with the APP catalytic process (Guo et al., 2018). Furthermore, the presence of mutant PS1 has also been associated with decreased autophagosome-lysosome fusion, lysosomal dysfunction, and neuronal loss (Nah et al., 2015; Bar-Yosef et al., 2019). Autophagy is also responsible for the clearance of tau forms including the phosphorylated form (Nah et al., 2015). Alterations in the autophagy pathway delay tau clearance leading to its accumulation and formation of neurofibrillary tangles (Nah et al., 2015).

### 4.1. Macroautophagy in AD

Dysfunctional macroautophagy in AD has been observed at the autophagy induction level, autophagosome maturation, and trafficking of the autophagosome to the neuronal cell body as well as at the autophagosome-lysosome function level (Nah et al., 2015; Bar-Yosef et al., 2019). The first piece of evidence that autophagy may be involved in the pathophysiology of AD was the striking accumulation of immature autophagosome vesicles observed in dystrophic neurites in AD brain tissue suggesting impaired autophagy (Nah et al., 2015; Guo et al., 2018). Downregulation of beclin 1, a key component of the macroautophagy machinery at the autophagy induction phase, has been reported in AD brains (Nah et al., 2015; Guo et al., 2018). Another abnormality in macroautophagy in AD includes the cleavage of beclin 1 by caspase 3, a component of the apoptosis pathway disrupting autophagy (Guo et al., 2018). Alterations in autophagosome trafficking in AD contribute to the accumulation of autophagic vacuoles, also known as immature autophagic vacuoles, in neurons and prevents the autophagosome from reaching the lysosome for fusion and subsequent cargo degradation (Nah et al., 2015). Furthermore, the accumulation of immature autophagic vacuoles stimulates autophagy through AMPK activation, a well-known autophagy activation pathway, suggesting that autophagy overactivation is the result of autophagosome accumulation caused by reduced trafficking (Nah et al., 2015). Autophagosome trafficking is also affected by the decreased levels of beclin 1 (Nah et al., 2015). Disturbances at the autophagosome-lysosome fusion level have been observed in AD (Guo et al., 2018). For instance, PS1 participates in the process of autophagosome and lysosome fusion, and lack of phosphorylated PS1 interrupts this process resulting in decreased autophagosome-mediated clearance (Guo et al., 2018; Bar-Yosef et al., 2019). Aberrant PS1 can degrease the degradation of cargo causing the accumulation of autophagosomes in neurons (Guo et al., 2018; Bar-Yosef et al., 2019). Additionally, PS1 regulates the internal environment in the lysosome by promoting acidification and activation of cathepsins which are proteases responsible for proteolytic degradation inside the lysosome (Bar-Yosef et al., 2019). Recent evidence showed the connection between plaque formation and autolysosome acidification. Before extracellular AB deposition, a decreased in autolysosome acidification was observed in neurons which led to the build-up of AB and the pathogenic APP metabolite APP-βCTF that, in normal autophagy functioning, are delivered to lysosomes for degradation (Lee et al., 2022). In compromised neurons, autophagy vacuoles containing AB pack into large structures forming flower-like perikaryal rosettes known as PANTHOS (poisonous Anthos or flower) which occur in the brains of AD patients (Lee et al., 2022). Another key player in autolysosome acidification is SIRT5, a sirtuin protein found in the mitochondria (Baeken, 2023). SIRT5 mediates the deacetylation and activation of the enzyme lactate dehydrogenase B found on the lysosomal membrane supplying the lysosomes with protons for lysosomal acidification (Baeken, 2023). Due to both its role in lysosomal acidification and its decreased expression in AD, SIRT5 has been regarded as an attractive autophagy-related target in AD therapeutics (Baeken, 2023).

### 4.2. Microautophagy in AD

In microautophagy, cell membrane components are captured through the formation of the multivesicular body (MVB), or intracellular components are engulfed at the lysosomal membrane (Lata et al., 2009). A variety of complexes or proteins are involved in the process of MVB-mediated microautophagy, including endosomal sorting complex required for transport (ESCRT), glyceraldehyde-3-phosphate dehydrogenase, aldolase, and cyclophilin (Shpilka and Elazar, 2011). Lysosomes and endosomes can directly trap cytosolic cargo through the boundary membrane, and MVBs are directly involved in the regulation of intracellular lysosome and endosome formation. Therefore, multivesicular lysosomes and MVBs are always considered the major biomarkers of microautophagy (Li et al., 2012). Mutations of several microautophagy-related genes in the promotion of different neurodegenerative diseases have been reported. For example, charged MVB protein 2B (CHMP2B) is a subunit of ESCRT and mutation of CHMP2B could lead to the pathogenesis of amyotrophic lateral sclerosis (Parkinson et al., 2006) and frontotemporal dementia (Skibinski et al., 2005). Mutations of VPS4A, a dominant negative form of the CHMP2B-interacting protein in primary neurons, lead to accumulation and aggregation of Aβ and phosphorylation of tau, suggesting that dysfunction of microautophagy could be considered as a risk factor for the pathogenesis of AD (Willen et al., 2017). Additionally, microautophagy deficit may be implicated in the regulation of tau-induced AD. For instance, IST1 is a positive regulator for the formation of the ESCRT complex. Feng et al. (2020) reported that overexpression of IST1 reduces the accumulation and aggregation of tau, and ameliorates synaptic plasticity and cognitive functions in human tau transgenic mice (Feng et al., 2020).

### 4.3. Chaperone-mediated autophagy in AD

The presence of mutant tau proteins contributes to the formation of neurofibrillary tangles arising from mutant tau aggregation in AD (Cuervo and Wong, 2014). Tau contains CMA-target motifs in the C-terminal region that make it prone to interact with the chaperone protein Hsc70, which is responsible for the translocation of CMA substrates to the lysosome for degradation (Wang et al., 2009). In AD brain tissue, Hsc70 associates with neurofibrillary tangles indicating that the CMA system is also involved in the degradation of neurofibrillary tangles (Wang et al., 2009). Mutant tau variants are processed by the CMA degradation system in which mutant tau is bound to the LAMP2A receptor and only partially internalized resulting in the formation of smaller amyloidogenic tau fragments in the lysosome (Cuervo and Wong, 2014). The tau fragments generated oligomerize at the lysosomal surface interfering with the lysosomal membrane integrity and affecting CMA’s normal functioning (Cuervo and Wong, 2014). Lysosome rupture releases mutant tau oligomers that serve as seeds for subsequent tau aggregation in the cytosol aggravating the formation of neurofibrillary tangles (Cuervo and Wong, 2014). Another protein implicated in dysfunctional CMA in AD is the regulator of calcineurin 1 (RCAN1). RCAN1 undergoes CMA-mediated degradation and is highly expressed in AD brains (Cuervo and Wong, 2014). It has been suggested that pathogenic tau clocking the CMA pathway leads to elevated levels of RCAN1 in the neuron (Cuervo and Wong, 2014). However, the contribution of RCAN1 to AD phenotype is still not well understood (Cuervo and Wong, 2014). More interestingly, a novel CMA-activating drug, metformin, recently identified during a high-throughput screening, has been found to reduce Aβ protein levels and improve cognitive impairment in a mouse model of AD, via Hsc70 signaling (Xu et al., 2021). The Aβ protein precursor contains three putative KFERQ-like CMA motifs that are strongly conserved across species. Metformin-induced CMA activity and Aβ plaque degradation involved these motifs, as the results revealed that mutation of the three KFERQ-like motifs blocked Aβ degradation as well as inhibiting the interaction of Aβ precursor protein with endogenous Hsc70 or LAMP2A (Xu et al., 2021).

### 4.4. Autophagy *in vitro* methods in AD research

Table 2 shows a list of cell-based approaches used in autophagy research in AD. The methods used to study the mechanism of selective (e.g., mitophagy) and non-selective macroautophagy focus on cargo recognition, induction, autophagosome flux, autophagosome maturation, and autolysosome formation stages as well as lysosomal function. *In vitro* platforms to study CMA in AD models mostly focus on the induction phase and the mechanism of cargo recognition and translocation while the formation of late endosomes and MVBs is assessed in microautophagy *in vitro* studies. Several AD *in vitro* models have been generated to manipulate the gene expression of relevant key molecular players, to assess the activity of key enzymes involved in the pathophysiology of AD, and to monitor substrate degradation in the presence and absence of autophagy inhibitors and activators.

**Table 2:** *In vitro* autophagy methods in Alzheimer’s disease research.

Autophagy mechanism	Autophagy stage	Molecular target	Cell line	Application (A), description (D), and readout (R)	References
Selective MA (mitophagy)	Cargo recognition, autophagic flux, and autophagosome maturation	Amyloid-β (Aβ) peptides	SH-SY5Y cells Primary neurons	**A**: Identification of mitophagy triggered by amyloid-β (Aβ) oligomers. **D**: Cells were treated with Aβ oligomers in the presence and absence of the inhibitor of autophagic vacuole maturation bafilomycin. The co-localization of the autophagy markers p62 and LC3 with a mitochondria marker was identified as an indication of induced mitophagy. **R**: (1) Protein expression by Western blot and detection of p62 and LC3 by immunofluorescence. (3) Calculation of the amount of colocalized mitochondria by Velocity software.	de la Cueva et al. (2022)
MA	Induction, autophagosome maturation, and lysosomal function	Presenilin 1 (PS1) mTOR	Murine blastocysts expressing different PS1 genotypes	**A**: Identification of loss of MA through deletion of PS1 by engineering cells and using MA activators and inhibitors. **D**: Cells with different PS1 genotypes including wild-type cells, PS1 knockout, mutant PS2, and wild-type human PS1 were treated to identify the role of PS1 in lysosome acidification and function. Autophagic/lysosomal degradation was induced through serum withdrawal, proteolysis was blocked using ammonium chloride, and MA was induced using rapamycin and inhibited using 3-methyladenine. **R**: (1) Classification and quantitation of autophagy vacuoles by ultrastructural and morphometric analysis using electron microscopy. (2) Protein degradation measured by pulse-chase tracking [^3^H]-leucine incorporation into proteins. (3) Protein phosphorylation by Western blot targeting p70S6 kinase and its phosphor-epitope to identify mTOR-mediated autophagy induction. (4) Protein expression by immunostaining targeting LC3, Western blot of LC3I and L3CII, and immunofluorescence targeting LC3 and LAMP2A.	Lee et al. (2010)
MA	Induction	Beclin 1	Ba/F3 cells	**A**: Identification of beclin 1 as a substrate of caspases as a cause of lower levels of beclin 1 in AD. **D**: Use of a site-directed caspase-cleavage antibody targeting beclin 1 to assess whether beclin 1 proteolytic cleavage by caspases is responsible for the lower levels of beclin 1 in AD. Detection of a 35 kDa C-terminal fragment of beclin 1 by a novel antibody is followed by incubation with caspase-3 to detect caspase-mediated cleavage of beclin 1. Cells were engineered to express two mutant beclin 1 forms expressing potential caspase-cleavage sites. **R**: (1) Protein expression analysis by Western blot targeting a caspase-resistant form of beclin 1.	Rohn et al. (2011)
MA	Induction	mTOR	7PA2 cells and Chinese hamster ovary cells were used as control	**A**: Assessment of autophagy induction using cells engineered to express an AD mutation that leads to the production of high levels of Aβ oligomers. **D**: mTOR phosphorylation was monitored to assess the induction of autophagy in both cell types. Autophagy activation mediated by Aβ and amyloid β-precursor protein was evaluated using the γ-secretase inhibitor compound E. mTOR inhibition using rapamycin was done to assess the effects of mTOR function on the levels of Aβ. **R**: (1) Protein expression by Western blot targeting mTOR, p-mTOR to assess autophagy induction. (2) Levels of intracellular Aβ measured by ELISA.	Caccamo et al. (2010)
MA	Autophagy induction, autophagic flux, and autolysosome formation	mTOR, LC3II	Primary cortical neurons	**A**: Evaluation of autophagosome and autolysosome formation using cells engineered to label LC3. Also, evaluation of protease activity and vesicle acidification using a fluorescent probe (BODIPY FL pepstatin A) to inhibit cathepsin D in lysosomes. **D**: Cells were treated with rapamycin to induce autophagy and measure mTOR-mediated autophagy activation. Labeled LC3 and BODIPY-pepstatin-FL labeling were used to assess autophagosome formation and maturation. **R**: (1) Protein expression analysis by Western blot targeting mTOR, p-mTOR, LC3I, and LC3II and immunohistochemistry to detect LC3. (2) Evaluation of autophagosomes and autolysosomes by morphometric analysis using confocal microscopy.	Boland et al. (2008)
CMA	Induction and cargo translocation	Hsc70, Aβ proteins	HEK293T and H4 cells	**A**: Evaluation of Aβ protein degradation mediated by Hsc70 and LAMP2A using engineered cells. **D**: HEK293T cells were engineered to express HK2-GFP in a doxycycline (DOX)-inducible manner (293THK). Cells were treated with the autophagy inhibitors Spautin-1 and Fms-like tyrosine kinase-3 (Flt3) inhibitor AC220 to induce CMA-mediated degradation of HK2. Hsc70 and LAMP2A were knockdown to assess CMA activation and monitor HK2 degradation. **R**: (1) Protein degradation by immunofluorescence to assess CMA-mediated degradation of HK2. (2) Gene and (3) protein expression of Hsc70, p-Hsc70, HK2 by qRT-PCR and Western blot, respectively.	Xu et al. (2021)
CMA	Induction, cargo recognition, and translocation	Hsc70, LAMP2A, and amyloid-beta precursor protein (APP)	SH-SY5Y cells	**A**: Monitoring the degradation of APP in a Hsc70 and LAMP2A-dependent manner by cell engineering to flag Hsc70 and LAMP2A. **D**: Cells were treated with a CMA inducer and lysosomal inhibitors such as E-64D, bafilomycin A1 (BafA1), leupeptin combined with ammonium chloride, and the protease inhibitor MG132 to assess lysosome-dependent degradation, Hsc70 phosphorylation, and APP degradation. **R**: (1) Protein expression by Western blot targeting Hsc70, pHsc70, LAMP2A, and APP.	Xu et al. (2021)
MiA	Late endosomes/MVBs	Aβ	Primary neuronal cells	**A**: Identification of the overexpression of VPS4 can lead to Aβ accumulation in enlarged endocytic compartments. **D**: Overexpression of VPS4 increased the intracellular pool of Aβ and decreased the amounts of Aβ secreted into the medium. **R**: (1) Protein expression by Western blot analysis targeting Aβ and by immunocytochemistry targeting Aβ.	Willen et al. (2017)

MA, macroautophagy; MiA, microautophagy; CMA, chaperone-mediated autophagy.

Cell-based methods that mimic AD hallmarks (e.g., over production of Aβ oligomers) are particularly useful in AD research. A mutation known as 7PA2, an APP isoform containing Val → Phe mutation at residue 717, can be introduced to Chinese hamster ovary cells to generate 7PA2 cells that overproduce Aβ oligomers (Portelius et al., 2012). The characteristics of the 7PA2 cell model make it a first-choice model to study Aβ-induced toxicity, a physiological feature relevant to the pathogenesis of AD (Portelius et al., 2012). Macroautophagy induction can be triggered by the presence of high levels of Aβ in 7PA2 cells (Caccamo et al., 2010). In this cell model, the role of autophagy key signaling such as the mTOR pathway could be monitored in function of Aβ levels when treating the cells with autophagy activators and inhibitors (Caccamo et al., 2010). At the autophagosome level, primary cortical neurons can be engineered to express LC3 tagged with a green fluorescent protein (GFP) to monitor autophagic flux by confocal microscopy (Boland et al., 2008). To assess autolysosome formation and function in the same type of modified cells, the fluorescent probe BODIPY FL pepstatin A is used to monitor protease activity and vesicle acidification through the inhibition of cathepsin D, a protease found in the lysosome that participates in the APP degradation (Chen et al., 2000; Boland et al., 2008; Uddin et al., 2018). A key molecular player in AD is PS1, a protein involved in the cleavage of APP, as the presence of mutant forms of PS1 not only disrupts APP catalysis but also interrupts the macroautophagy process at different stages (e.g., autophagosome-lysosome function) (Guo et al., 2018; Bar-Yosef et al., 2019). The genetic manipulation of PS1 in *in vitro* models is useful to identify the effect of PS1 in macroautophagy. For instance, murine blastocysts can be engineered to express different PS1 genotypes, including PS1 knockout, mutant PS1, and wild-type human PS1, to assess the PS1 effect at the lysosome level using ultrastructural and morphometric analysis by electron microscopy to analyze autophagy vacuoles (Lee et al., 2010). An *in vitro* method to study mitophagy, a selective macroautophagy mechanism, focuses on the assessment of cargo recognition, autophagic flux, and autophagosome maturation in SH-SY5Y cells and primary neurons (de la Cueva et al., 2022). In this model, cells are challenged with AB oligomers in the presence and absence of the inhibitor of autophagic vacuole maturation BafA1 (de la Cueva et al., 2022). The colocalization of the autophagy markers p62 and LC3 with the mitochondrial marker CxVβ allows monitoring of the mitophagy process using immunofluorescence and Western blot analysis as well as the quantitation of colocalized mitochondria by immunofluorescence (de la Cueva et al., 2022).

A useful approach to studying CMA combines the downregulation of pivotal key players in CMA such as Hsc70 and LAMP2A with overexpression of CMA substrates such as hexokinase 2 (HK2), an enzyme that participates in glucose metabolism, which is targeted by the CMA machinery for degradation (Xu et al., 2021). This reporter system is CMA-specific and suitable for high-throughput screening and is characterized by HEK293 cells modified to express the HK2-GFP complex in doxycycline (DOX), a second-generation tetracycline that activates autophagy,-inducible manner to obtain 293THK cells (Xing et al., 2017; Xu et al., 2021). The system allows the identification of CMA activation and substrate degradation using immunofluorescence, Western blot, qRT-PCR, and flow cytometry (Xu et al., 2021). APP degradation as well as CMA induction, cargo recognition and translocation can be assessed in SH-SY5Y cells expressing Hsc70- and LAMP2A-Flag treated with CMA inducers and lysosomal inhibitors such as E-64D, BafA1, leupeptin, ammonium chloride, and the protease inhibitor MG132 in combination with the detection of the accumulation of AB using immunocytochemistry and Western blot (Xu et al., 2021).

A cell-engineering approach to generate an AD model to study the mechanism of microautophagy involves primary neuronal cells that overexpress VPS4A (Willen et al., 2017). VPS4A is an enzyme that regulates endosomal sorting complexes (ESCRTs) responsible for membrane modeling and topology processes such as the formation of late endosomal MVBs (Willen et al., 2017; Rodger et al., 2020). Overexpression of VPS4A mimics AD hallmarks such as the accumulation and aggregation of AB in MVBs and enlarged late endocytic compartments (Willen et al., 2017). In this *in vitro* system, the intra and extracellular accumulation of AB is monitored to assess late endosome and MVB formation (Willen et al., 2017).

## 5. Amyotrophic lateral sclerosis (ALS)

ALS is an age-dependent neurodegenerative disease characterized by the selective degeneration of motor neurons in the brain and spinal cord (Guo et al., 2018; Amin et al., 2020). The clinical manifestation of ALS includes progressive muscle weakness, muscle wasting, atrophy, and paralysis starting in the distal muscles of limbs moving toward proximal muscles as the disease progresses (Masrori and Van Damme, 2020). Most ALS cases manifest between 50 and 60 years of age and culminate in death caused by respiratory failure (Amin et al., 2020). Accumulation of protein aggregates and mitochondrial damage are responsible for motor neuron degeneration and death in ALS (Chen et al., 2012). It is estimated that about 90% of ALS cases are sporadic and 10% are associated with genetic mutations (Nguyen et al., 2019). The hallmark of ALS is the cytoplasmic aggregation of TDP-43 protein, which is observed in more than 95% of ALS cases (Amin et al., 2020). Furthermore, about 20% of the classic familiar ALS cases are attributed to mutations in SOD1 (Chen et al., 2012). Other genes associated with ALS include fused in sarcoma (FUS), sequestosome1 (SQSTM1 also known as p62), optineurin (OPTN), TANK binding kinase 1 (TBK1), VAMP-associated protein B (VAPB), valosin-containing protein (VCP), ubiquilin 2 (UBQLN2), alsin, charged multivesicular body protein 2B (CHMP2B), dynactin (DCTN), profilin 1 (PFN1), factor-induced gene 4, and a hexanucleotide repeat expansion in the gene C9orf72 (Guo et al., 2018; Nguyen et al., 2019). The accumulation of mutant SOD1 aggregates and autophagic vacuoles in motor neurons of the spinal cords points out at aberrant autophagic flux or specific dysfunctional autophagy regulatory processes that may lead to motor neuron degeneration and death (Chen et al., 2012). Mutant SOD1 can be recognized by ubiquitin-interacting proteins such as p62 for selective autophagy clearance, but this mutant protein can inhibit the autophagy pathway leading to decreased clearance (Chen et al., 2012; Nguyen et al., 2019). Moreover, mutant SOD1 alters the microtubule-localized dynein-dynactin complex affecting autophagosome trafficking (Nguyen et al., 2019). Alterations in autophagosome maturation and fusion with the lysosome have also been attributed to the presence of mutant SOD1 (Chen et al., 2012). For instance, mutant SOD1 leads to the accumulation of mitochondria and membrane-bound organelles by disruption of the axonal transport (Chen et al., 2012). Ubiquitinated aggregates in ASL are mainly formed by the accumulation of abnormally modified and cleavage forms of TDP-43 that cannot be efficiently cleared by either autophagy or the proteasome system (Chen et al., 2012). Aberrant TDP-43 impairs its ubiquitination leading to dysfunctional protein clearance and destabilizing Atg7 mRNA impairing autophagosome formation (Amin et al., 2020). Mutant forms of other proteins also alter the autophagy machinery at different stages. For example, mutant FUS, VAPB, C9orf72, and CHMP2B affect autophagosome formation, mutant OPTN, TBK1, CHMP2B, PFN1, and DCTN impair retrograde transport for autolysosome formation, mutant C9orf72, OPTN, and CHMP2B disrupt autolysosome formation, and mutant p62, OPTN, and UBQLN2 affect cargo degradation (Amin et al., 2020).

### 5.1. Macroautophagy in ALS

Dysregulation at different macroautophagy stages, particularly at induction, trafficking, and autophagosome-lysosome fusion, has been implicated in ALS (Guo et al., 2018; Amin et al., 2020). For instance, increased levels of LC3, beclin 1, p62, and the Atg5-Atg12 complex, which are mediators involved in the early stages of the macroautophagy process, have been detected in spinal motor neurons of both sporadic and familial ALS that later extend to other cells such as astrocytes and microglia (Amin et al., 2020). Aberrant accumulation of immature autophagosomes observed in spinal motor neurons suggest alterations of the macroautophagy machinery at autophagosome maturation and subsequent lysosomal function level in which mutant forms of VAPB, UBQLN2, VCP, PC2, OPTN, and TBK1 proteins have been implicated (Guo et al., 2018; Amin et al., 2020). The dynein-dynactin complex is responsible for the retrograde transport of autophagosomes (Nguyen et al., 2019). Autophagosomes mature during transportation as a result of acidification that occurs before lysosomal fusion when the autophagosome reaches the cell soma (Nguyen et al., 2019). However, misfolded mutant SOD1 is prone to increase its interaction with dynein leading to an overload of dynein-mediated retrograde axonal transport which in turn affects autophagosome trafficking (Nguyen et al., 2019). At autophagosome and lysosome fusion levels, aberrant autophagy regulators such as C9orf72, VCP, CHMP2B, ALS2, and (Table 3) affect the formation of the autolysosome leading to reduced cargo degradation and build-up of autophagosomes and aberrant proteins in motor neurons and glial cells (Amin et al., 2020).

**Table 3:** *In vitro* autophagy methods in amyotrophic lateral sclerosis research.

Autophagy mechanism	Autophagy stage	Molecular target	Cell line	Application (A), description (D), and readout (R)	References
MA, selective MA (mitophagy), and CMA	Induction, autophagic flux, and autophagosome-lysosome fusion	SOD1, TDP-43, LC3, p62, LAMP2A, and Hsc70	NSC-34 cells Motor neurons differentiated from H9 human embryonic stem cells (hESCs)	**A**: Monitoring autophagy at different stages using cells expressing mutant SOD1 and mutant TDP-43 and validation in hESC-derived spinal motor neurons. **D**: NSC-34 cells expressing mutant SOD1 and TDP-43 were treated with the autophagy activator rilmenidine and autophagy inhibitors bafilomycin A1 (BafA1) and rotenone. MA and CMA markers, autophagic flux, were evaluated to assess autophagy induction. NSC-34 cells were engineered to obtain a mCherry-GFP-LC3B reporter system to assess autophagic flux. The fusion of the mitochondria with lysosomes was analyzed to assess mitophagy in cells engineered with the ratiometric pHluorin DsRed-T3 to evaluate the percentage of DsRed-T3-positive mitochondria fused with lysosomes. hESC-derived spinal motor neurons were engineered to express wild-type SOD1 and treated with an autophagy inducer. **R**: (1) Protein expression by Western blot targeting LC3I and LC3II, p62, LAMP2A, and Hsc70. (2) Autophagosome and autolysosome fusion by confocal microscopy analysis of mature autolysosomes. (3) Fusion of the mitochondria with lysosomes by confocal microscopy to identify DsRed.T3-positive mitochondria. (4) Autophagic flux using a tandem mCherry-GFP-LC3B reporter construct to identify mature autolysosomes.	Perera et al. (2017)
MA	Substrate degradation	SOD1 and TDP-43	NSC-34 cells	**A**: Identification of SOD1 and TDP-43 aggregates in cells overexpressing mutant SOD1. **D**: Cells were engineered to overexpress SOD1 and treated with the autophagy inductor trehalose. The presence of SOD1 and TDP-43 aggregates was assessed. **R**: (1) Protein expression by Western blot targeting SOD1 and immunofluorescence targeting TDP-43 and ubiquitin.	Gomes et al. (2010)
MA	Autophagic flux, substrate degradation	LPD-43, LPD-25, LC3	HEK293 cells	**A**: Monitoring the degradation of TDP-43 in TDP-43 and TDP-25 overexpressing cells. **D**: Cells were engineered to overexpress TDP-43 and TDP-25 in a green fluorescence protein system. Then, cells were treated with the autophagy inducer trehalose and autophagy inhibitor 3-methyladenine. Cells were further modified to tag LC3 with a red fluorescent protein and treated with the same autophagy inducer and inhibitor to assess MA-mediated substrate degradation. **R**: (1) Protein expression by Western blot, immunofluorescence, and immunoprecipitation targeting TDP-43, TDP-25, and ubiquitin.	Wang et al. (2010)
CMA	Substrate recognition	CMA recognition motif by Hsc70	N2a cells	**A**: Evaluation of TDP-43 degradation through the CMA system by modifying the CMA recognition motif in TDP-43. **D**: Cells were modified to express a mutation in the KFERQ-like motif of TDP-43. The interaction of the mutant TPD-43 and Hsc70 was assessed. Cells were treated with the lysosome inhibitor ammonium chloride and 3-methyladenine (3-MA). Also, LAMP2A was knocked down to assess CMA-mediated degradation. **R**: (1) Protein expression by Western blot and immunofluorescence targeting TDP-43, TDP-35, TDP-25, LAMP2A, and Hsc70.	Huang et al. (2014)
CMA	Substrate degradation	Hsc70 expression and TDP-43	Primary lymphomonocytes of sporadic ALS patients, lymphoblastoid cell lines derived from sporadic ALS patients, and SH-SY5Y cells	**A**: Monitoring the expression of TDP-43 and Hsc70 activity in an ALS model consisting of cells engineered to silence Hsc70 to assess CMA-mediated degradation of TDP-43. **D**: Peripheral blood mononuclear cells from sporadic ALS patients were used to obtain immortalized lymphoblastoid cells. The expression of Hsc70, LAMP2A, Bag1, Bag3, and LPD-43 was measured in both cell lines to assess CMA and LPD-43 accumulation. The expression of Hsc70 was downregulated in SH-SY5Y cells to assess the participation of Hsc70 in TDP-43 degradation. **R**: (1) Gene and (2) protein expression assessed by qRT-PCR and Western blot, respectively, targeting Hsc70, LAMP2A, and co-chaperones Bag1 and Bag3. Substrate degradation was evaluated by immunofluorescence analysis targeting TDP-43. (3) Identification of insoluble TDP-43 by filter retardation assay using a Bio-Dot SF microfiltration apparatus and Dot Blot analysis.	Arosio et al. (2020)

MA, macroautophagy; CMA, chaperone-mediated autophagy.

### 5.2. Microautophagy in ALS

The relationship between microautophagy and ALS is rarely reported. However, a study reported that mutation of CHMP2B can lead to ALS pathogenesis (Parkinson et al., 2006), suggesting the potential role of microautophagy. Additionally, in Drosophila models of ALS and human neuronal cells, scientists found that overexpression of TAR DNA-binding protein 43 (TDP-43) can inhibit HSPA8 transcription, leading to the dysfunction of synaptic vesicle cycling all of which is associated with microautophagy dysfunction (Coyne et al., 2017).

### 5.3. Chaperone-mediated autophagy in ALS

The involvement of CMA in ALS is quite poorly understood despite the insight into the contribution of dysfunctional autophagy at large in the pathogenesis of the disease (Lee et al., 2015). Of the many ALS-associated protein aggregates, TDP-43 is the most studied in terms of its correlation with CMA activity (Liao et al., 2021). It has been found that the TDP-43 protein contained the CMA-recognition motif sequence Q^134^VKKD^138^, a KFERQ-like motif, suggesting that TDP-43 could be a substrate for CMA (Huang et al., 2014). Mutation of the KFERQ-like motif in the TDP-43 coding gene inhibited the CMA degradation of TDP-43 and led to the aggregation of TDP-43. By immunoprecipitation, it was demonstrated that TDP-43 proteins with the KFERQ-like motif in transfected N2a cells interacted with the molecular chaperone Hsc70, which facilitated the degradation of the toxic protein in cells with macroautophagy inhibition (Huang et al., 2014). Besides the TDP-43 protein, the regulatory role of CMA in other ALS-associated toxic mutant proteins, such as SOD1 found in familial forms of ALS, has not yet been explicitly shown (Crippa et al., 2010).

### 5.4. Autophagy *in vitro* methods in ALS research

A summary of cell-based platforms used in ALS research is presented in Table 3. Most studies target substrate degradation, autophagy induction, autophagic flux, and autophagosome-autolysosome fusion in macroautophagy and substrate recognition and degradation in CMA (Table 3). Crucial molecular features of ALS pathophysiology include the cytoplasmatic aggregation of TDP-43 and mutant SOD1 that are responsible for the disruption of the autophagy machinery in motor neurons of the spinal cord (Chen et al., 2012; Amin et al., 2020). Most cell-based methods in ALS research are therefore concerned with the role of TDP-43 and SOD1 at different stages of the mechanism of autophagy.

In several *in vitro* models to study selective and non-selective macroautophagy as well as CMA, cells are engineered to express mutant SOD1 and TDP-43. For instance, the spinal cord neuroblastoma hybrid cell line NSC-34, a cell line of choice in ALS research, has been modified to express mutant form SOD1 and TDP-43 (Perera et al., 2017; Amin et al., 2020). NSC-34 cells expressing those mutant proteins are then challenged with autophagy activators and inhibitors (e.g., rilmenidine, BafA1, or rotenone) to assess macroautophagy, mitophagy, and CMA at different stages of the autophagy-mediated degradation process (Perera et al., 2017). By applying traditional (e.g., Western blot) and more sophisticated analytical techniques such as gene reporter assays (e.g., DsRed-T3 or mCherry-GFP-LC3B reporter assays), key autophagy players (e.g., LC3I, LC3II, P62, LAMP2A, and Hsc70) can be monitored to assess aberrant autophagy and the effect of the presence of mutant proteins relevant to ALS (Perera et al., 2017). For instance, the mCherry-GFP-LC3B reporter system is a tandem system to identify mature autolysosomes and measure autophagic flux by confocal microscopy (Perera et al., 2017). Furthermore, the fusion of mitochondria with the lysosome can be monitored using a ratiometric pHluorin DsRed-T3 system (Perera et al., 2017). In this system, the mitochondria are labeled, and the presence of labeled mitochondria in lysosomes can be used as an indication of the successful fusion of the mitochondria-containing autophagosome with the lysosome (Perera et al., 2017). A variation of the cell-based approach mentioned-above in NSC-34 cells involves monitoring the degradation of SOD1 and TDP-43 aggregates in cells overexpressing mutant SOD1 (Gomes et al., 2010). Upon autophagy induction, the degradation of SOD1, TDP-43 aggregates, and ubiquitinated TDP-43 can be analyzed by Western blot and immunofluorescence (Gomes et al., 2010). TDP-43 C-terminal fragments such as TDP-25 and TDP-35 exhibit pathogenic properties as observed in diseased brains (Wang et al., 2010). These TDP-43-derived fragments of lower molecular weight are also prone to aggregation and worth attention in cell-based platforms (Wang et al., 2010). A cell model to monitor the degradation of TDP-43 fragments through macroautophagy involves HEK293 cells engineered to overexpress TDP-43 and TDP-25 using a green fluorescent protein system (Wang et al., 2010). Using autophagy inhibitors and activators, autophagic flux and substrate degradation are evaluated by Western blot, immunofluorescence, and immunoprecipitation (Wang et al., 2010).

*In vitro* platforms focusing on the CMA degradation process monitor TDP-43 recognition using cell systems that modify the TDP-43 site in a fast-growing mouse neuroblastoma cell line, N2a (Huang et al., 2014). In this cell model, N2a cells are engineered to modify the KFERQ-like motif recognition site present in TDP-43 leading to assessing the interaction of this aberrant protein with Hsc70 for substrate recognition in CMA (Huang et al., 2014). The use of lysosome inhibitors (e.g., 3-MA and ammonium chloride) and cells with downregulated expression of LAMP2A in parallel with the analysis of TDP-43-derived fractions and the CMA key players LAMP2A and Hsc70 are also approaches to assess the CMA degradation of TDP-43 (Huang et al., 2014). Other systems targeting TDP-43 degradation by CMA use patient-derived cells and cells in combination with other cell lines commonly used in neurodegeneration research such as SH-SY5Y cells (Arosio et al., 2020). For instance, the accumulation of TDP-43 as well as gene and protein expression of CMA markers can be evaluated in immortalized lymphoblastoid cells obtained from peripheral blood mononuclear cells isolated from sporadic ALS patients (Arosio et al., 2020). A filter retardation assay using a Bio-Dot SF microfiltration system to conduct Dot Blot analysis can be employed to measure the presence of insoluble TDP-43 (Arosio et al., 2020). Downregulation of Hsc70 in SH-SY5Y cells is a useful approach to evaluate the role of Hsc70 in TDP-43 degradation (Arosio et al., 2020).

## 6. Huntington’s disease (HD)

First described by George Huntington in 1872, HD is an autosomal dominant progressive neurodegenerative disorder characterized by loss of motor control, cognitive impairment, bradykinesia, rigidity, muscle wasting, weight loss, and eventually death (Nah et al., 2015; Bar-Yosef et al., 2019; Stoker et al., 2022). Other symptoms in HD include ticks and impaired gait and postural stability that manifest as a result of dystonia, rigidity, chorea, and ataxia (Stoker et al., 2022). The onset of symptoms occurs between 35 and 50 years (Bar-Yosef et al., 2019). The prevalence of HD is 10.6–13.7 cases per 100,000 in Western populations while a lower prevalence, 1–7 cases per million, has been reported in Eastern populations such as Japan, Taiwan, and Hong Kong (McColgan and Tabrizi, 2018). HD is caused by the production of a mutant form of huntingtin protein (Nah et al., 2015). Huntingtin is a large scaffolding protein that has many interaction sites (Ghosh and Tabrizi, 2018). In HD, a mutation in huntingtin makes it interact with other proteins in an aberrant way leading to the disruption of cellular functions (Ghosh and Tabrizi, 2018). The presence of mutant huntingtin causes changes in its conformation that make it prone to form aggregates which affect several neuronal mechanisms such as cellular proteostasis, axonal transport, transcription, translation, mitochondrial and synaptic function and ultimately lead to neuronal death (Nah et al., 2015; Ghosh and Tabrizi, 2018; McColgan and Tabrizi, 2018). Mutant huntingtin is particularly damaging to medium spiny neurons of the striatum, which play a critical role in the regulation of signals from the cortex to output pathways, leading to the clinical manifestations observed in HD (Pan and Feigin, 2021). Although autophagy is the mechanism responsible for the clearance of mutant huntingtin, the presence of this aberrant protein affects the autophagy process (Nah et al., 2015). Therefore, the accumulation of aberrant huntingtin leads to inefficient autophagy contributing to HD pathogenesis (Nah et al., 2015).

### 6.1. Macroautophagy in HD

Alterations in the macroautophagy pathway and decreased macroautophagy-mediated clearance have been associated with the pathogenesis of HD (Bar-Yosef et al., 2019). Macroautophagy contributes to the elimination of mutant huntingtin aggregates, a process also known as aggregophagy (Croce and Yamamoto, 2019). Although the mechanisms for macroautophagy-mediated clearance of aggregates remain elusive, key mediators such as beclin 1, p62, Nbr1, Optn, and Alfy have been implicated in the mechanism of mutant huntingtin aggregate degradation (Croce and Yamamoto, 2019). At the cargo recognition level, mutant huntingtin interacts with p62 in HD cells causing problems in efficiently recognizing and engulfing the cytosolic cargo for degradation leading to the accumulation of toxic cellular components (Nah et al., 2015). In the normal cargo recognition process, the cargo gets sequestered by p62 binding to Atg8 cytosolic orthologues to bring the emerging autophagy membrane to the aggregate structure (Croce and Yamamoto, 2019). Other proteins such as LC3B and Alfy are also needed at this stage. Alfy binds to p62-tagged inclusions and the complex formed by the autophagy proteins Atg5, Atg12, and Atg16 to facilitate the conjugation of Atg8 proteins to the autophagosome membrane (Croce and Yamamoto, 2019). Furthermore, the beclin 1-complex involved in the autophagy initiation phase is affected because beclin 1 gets recruited by mutant huntingtin aggregates (Nah et al., 2015). In turn, the lack of autophagy initiation causes further accumulation of mutant huntingtin leading to neuronal death (Nah et al., 2015). The continuous accumulation of aggregates as a result of decreased macroautophagy-mediated clearance activates cell death pathways through apoptosis or autophagy leading to a dramatic loss of cells (Bar-Yosef et al., 2019).

### 6.2. Microautophagy in HD

One particular form of microautophagy, RN/DNautophagy, a degradation mechanism responsible for the direct uptake of RNA and DNA into lysosomes, has been reported as closely implicated in HD (Martin-Aparicio et al., 2001; Wang et al., 2023). Specifically, SID1 transmembrane family member 2 (SIDT2) can bind to exon 1 of the polyglutamine-expanded huntingtin, one of the hallmarks of HD pathology (Martin-Aparicio et al., 2001). Overexpression of SIDT2 can lead to the degradation of huntingtin mRNA and further reduce the level of the huntingtin complex (Hase et al., 2020). Currently, the biological functions of microautophagy and its role in the pathogenesis of HD are rarely reported.

### 6.3. Chaperone-mediated autophagy in HD

The chaperone Hsc70 is highly selective in recognizing the KFERQ-like motif in a protein substrate destined for degradation by CMA (Kirchner et al., 2019). Hence, the direct identification of KFERQ-like motifs in protein aggregates indicates the compromise of the CMA. The normal huntingtin protein has two KFERQ-like motifs: one at amino acid 99–103 (KDRVN), and the other at 248–252 (NEIKV). The 99-KDRVN-103 motif in the huntingtin protein functionally interacts with Hsc70, resulting in the delivery of the huntingtin protein to lysosomes for degradation by CMA (Fred Dice, 1990). In HD, the mutant huntingtin protein contains abnormally extended polyglutamine (polyQ) repeats, which are prone to fragmentation, misfolding, and aggregation in the basal ganglia and cortex (Cortes and La Spada, 2014). The polyQ-expanded mutant huntingtin fragments are also degraded by CMA, except that its degradation is relatively much slower leading to protein aggregation (Qi et al., 2012). The mutant huntingtin protein contained KFERQ-like motifs, and by manipulating the Hsc70 and LAMP2A levels, it was possible to promote mutant huntingtin degradation through CMA (Qi et al., 2012). The huntingtin-552 (Htt-552) overexpression model of HD was generated by adenoviral transfection of PC12 cells. The role of CMA in the degradation of Htt-552 was determined by overexpression or silencing of Hsc70 and LAMP2A. While the above evidence may suggest a decline in CMA activity in HD, rather studies have reported the constitutive upregulation of CMA in HD (Koga et al., 2011; Choi and Cho, 2021). CMA activity increases as a compensatory response to impaired macroautophagy in the early stages of HD. In cellular and mouse models of HD, LAMP2A, and Hsc70 were markedly increased (Koga et al., 2011). In another HD model with extended polyQ in striatal neurons, LAMP2A levels became elevated, inducing neuroinflammation. These results suggest that LAMP2A related to CMA capacity might play an important role in HD onset and progression (Choi and Cho, 2021). Thus, these pieces of evidence that (1) levels of mutant huntingtin fragments could be reduced by the upregulation of the CMA biomarkers LAMP2A and Hsc70; and (2) the observation of spontaneous changes in these biomarkers as the disease progresses give a strong justification to link CMA with HD.

### 6.4. Autophagy *in vitro* methods in HD research

The hallmark in HD is the presence of mutant huntingtin that is prone to aggregation disrupting crucial cellular processes that are relevant for neuronal survival (Ghosh and Tabrizi, 2018). Because of the damaging nature of mutant huntingtin aggregates, HD cell-based methods primarily focus on monitoring the autophagy-mediated molecular mechanisms of mutant huntingtin degradation. A selection of cell-based methods used to investigate autophagy in HD is shown in Table 4. *In vitro* HD models mainly target the mechanism of macroautophagy and CMA.

**Table 4:** *In vitro* autophagy methods in Huntington’s disease research.

Autophagy mechanism	Autophagy stage	Molecular target	Cell line	Application (A), description (D), and readout (R)	References
MA	Autophagic flux and substrate interaction with MA markers	Huntingtin, LC3II, LCI, and p62	Primary cells (embryonic fibroblasts from wild type, YAC128, and C6R mice) and COS-7 cells	**A**: Monitoring the interaction of mutant huntingtin with autophagy markers in primary cells engineered to overexpress mutant huntingtin and assessment of autophagic flux. **D**: Primary cells derived from mouse cell models were generated to determine the effect on autophagy of cleavable or C6R mutant huntingtin. Cells were treated with bafilomycin A1 (BafA1) to inhibit autophagic flux. Cells were modified to express a series of truncation mutants and treated with BafA1 to assess the interaction between mutant huntingtin and p62. Cos-7 cells were modified to express cleavable or a C6R mutant huntingtin form and p62 to assess huntingtin degradation. **R**: (1) Protein expression by Western blot (targeting LC3II, LC3I, and p62) and immunostaining (LC3 and p62) to measure the expression of autophagy markers. (2) Gene expression of p62 was evaluated by qRT-PCR. (3) Protein–protein interaction (mutant huntingtin forms and p62) by co-immunoprecipitation.	Ehrnhoefer et al. (2018)
MA	Induction, autophagic flux, and substrate degradation	Huntingtin, Atg4B, LC3II, LC3I, p62	Medium-sized-spiny neurons and cortico-striatal slice cultures	**A**: Monitoring of autophagy induction and identification of Atg4b-dependent autophagic flux in cells prone to aggregation of mutant huntingtin and engineered to overexpress Atg4B. **D**: Cells were treated with an mTOR inhibitor (AZD8055) and a lysosomal inhibitor (BafA1) to evaluate the accumulation of mutant huntingtin. A tandem fluorescent-tagged mCherry-GFP-LC3 system was used to monitor autophagic flux in the presence of BafA1. **R**: (1) Protein expression by Western blot targeting, LC3II, LC3I, Atg4B, and p62. (2) Substrate accumulation by immunohistochemistry and Western blot analyses targeting mutant huntingtin. (3) Autophagic flux by immunofluorescence (LC3).	Proenca et al. (2013)
MA and CMA	Induction, cargo recognition, and degradation	Huntingtin, LAMP2A, and Hsc70	Human lymphoblast from healthy or HD patients, striatal neurons from HD94 mice, primary ventromedial neurons, and mouse embryonic fibroblasts	**A**: Monitoring of mutant huntingtin degradation through CMA and MA using engineered and HD cells. **D**: Fibroblasts engineered to express mutant human huntingtin, as well as human lymphoblasts, and primary striatal cultures were treated with the autophagy inhibitors ammonium chloride and leupeptin to assess inhibition of lysosomal proteolysis relevant to the CMA pathway and 3-methyladenine to assess MA-mediated autophagy. CMA activity was assessed in control and HC cells using a photoswitchable reporter (KFERQ-PS-CFP2) to track cargo delivery to the lysosome. **R**: (1) Protein expression by immunofluorescence targeting mutant huntingtin. (2) Gene expression by qRT-PCR targeting LAMP2A and Hsc70. (3) Cargo delivery to the lysosome using the photoswitchable reporter KFERQ-PS-CFP2 to analyze intracellular immunofluorescence.	Koga et al. (2011)
CMA	Cargo recognition and degradation	LAMP2A, Hsc70, p26, and mutant huntingtin	Mouse striatal cell lines expressing mutant human huntingtin and STHdh cells	**A**: Assessment of the effect of let7b on CMA. **D**: Cells were modified to overexpress let7b and assess the effect of increased let7b on CMA by monitoring CMA markers. **R**: (1) Gene expression by qRT-PCR, and (2) protein expression by Western blot and immunocytochemistry targeting CMA markers and mutant huntingtin.	Choi and Cho (2021)
CMA and MA	Induction, cargo recognition, and degradation	KFERQ-like motifs in mutant huntingtin, CMA markers (LAMP2A and Hsc70), and the MA marker beclin 1	PC12 cells	**A**: Monitoring CMA through manipulation of the Hsc70 and LAMP2A levels in a cell model of HD generated by overexpression of mutant huntingtin. The contribution of MA degradation of mutant huntingtin was also assessed. **D**: Cells were engineered to overexpress mutant huntingtin and its degradation was monitored in cells showing overexpression and downregulation of the CMA markers Hsc70 and LAMP2A. Starvation was used to induce CMA. **R**: (1) Protein expression by immunofluorescence, immunoprecipitation, and Western blot analysis targeting LAMP2A, Hsc70, mutant huntingtin, and beclin 1. (2) Lysosomal integrity, purity, and content analysis were assessed using subcellular fractionation with a self-Percoll gradient followed by the analysis of the enzymatic activity of the lysosomal enzyme ß-hexosaminidase, and the detection of Hsc60, LAMP2A, huntingtin, and Hsc70 by Western blot.	Qi et al. (2012)

MA, macroautophagy; MiA, microautophagy; CMA, chaperone-mediated autophagy.

The most commonly used model is the overexpressing human mutant huntingtin in a wide variety of cells including embryonic fibroblasts, COS-7, medium sized-spiny neurons, cortico-striatal slice cultures, and PC12 cells (Koga et al., 2011; Qi et al., 2012; Proenca et al., 2013). To identify specific autophagy key players and autophagy stages that are crucial for mutant huntingtin degradation, cell-based methods are designed to measure the accumulation of mutant huntingtin after treatment with different autophagy inhibitors. For instance, accumulation of mutant huntingtin in cells treated with autophagy inhibitors such as the lysosomal proteolysis disruptors ammonium chloride and leupeptin and the autophagy induction inhibitor 3-MA can be monitored using immunofluorescence in both cells isolated from HD patients and mouse cells (Koga et al., 2011). Another cell system includes medium sized-spiny neurons and cortico-striatal slice cultures treated with the autophagy inhibitors AZD8055 and BafA1 followed by the evaluation of mutant huntingtin degradation by immunohistochemistry and Western blot (Proenca et al., 2013). Another approach to identifying the role of key autophagy players in mutant huntingtin degradation is to engineer PC12 cells to downregulate the expression of CMA markers Hsc70 and LAMP2A and measure the accumulation of mutant huntingtin by immunofluorescence, immunoprecipitation, and Western blot (Qi et al., 2012). Modifying cells to express mutant huntingtin and manipulating the expression of key autophagy mediators contribute to the mechanistic understanding of mutant huntingtin degradation. Cell engineering can be even extended to molecular players outside the autophagy machinery to identify potential therapeutic targets. For instance, mouse striatal cell lines expressing mutant human huntingtin and STHdh cells can be modified to overexpress the miRNA lethal-7b (let7b), a member of the let7 family responsible for the regulation of cell differentiation processes in neural stem cells (Choi and Cho, 2021; Chan et al., 2022). In this cell system, the role of let7 on CMA can be evaluated by monitoring CMA markers and mutant huntingtin accumulation in cells overexpressing let7b (Choi and Cho, 2021).

A critical step in mutant huntingtin degradation is substrate recognition by the autophagy machinery. Modifications can be added to mutant huntingtin to express a series of truncation mutants in primary and COS-7 cells (Ehrnhoefer et al., 2018). In this macroautophagy cell-based approach, the interaction of mutant huntingtin forms with p62 can be monitored by co-immunoprecipitation (Ehrnhoefer et al., 2018). In HD studies focusing on macroautophagy, autophagic flux is monitored using different techniques such as protein expression by Western blot targeting LC3II and LC3I, immunostaining of LC3, or using the novel tandem fluorescent-tagged mCherry-GFP-LC3 system (Proenca et al., 2013; Ehrnhoefer et al., 2018). In studies focusing on CMA autophagy in HD, traditional methods to monitor CMA markers such as Western blot or qRT-PCR are commonly applied (Koga et al., 2011; Qi et al., 2012; Choi and Cho, 2021). However, novel methods have been developed to assess CMA at the lysosomal level. For example, lysosomal integrity, purity, and analysis of the lysosomal content can be conducted using subcellular fractionation with a self-Percoll gradient followed by the analysis of the enzymatic activity of the lysosomal enzyme ß-hexosaminidase and detection of LAMP2A, huntingtin, and Hsc70 by Western blot (Qi et al., 2012). A recently developed method allows the evaluation of cargo delivery to the lysosome using a photoswitchable reporter KFERQ-PS-CFP2 system that can be tracked by analyzing intracellular immunofluorescence (Choi and Cho, 2021).

## 7. Depression

Major depressive disorder is a severe psychiatric illness that limits psychosocial functioning and diminishes the quality of life (Malhi and Mann, 2018). This multifactorial disorder significantly affects up to 10% of the global population and represents a tremendous economic burden to society (Jia and Le, 2015; Alcocer-Gómez et al., 2017). The lifetime risk of depression is high with estimations indicating that one in every five people experiences an episode of depression at some point in their lives (Malhi and Mann, 2018). The prevalence of depression is twice as common in women than in men with a peak in prevalence observed around the second and third decade of life (Malhi and Mann, 2018). Depression is characterized by depressed mood, anhedonia, feelings of worthlessness, low self-esteem, fatigue, changes in eating and sleeping patterns, and altered cognition (Alcocer-Gómez et al., 2017; Bar-Yosef et al., 2019). The pathogenesis of depression is still not well understood due to the complex multifactorial nature that involves both genetic and environmental factors (Jia and Le, 2015). Consequently, several hypotheses including the monoamine, hypothalamic–pituitary–adrenal (HPA) axis, neuroplasticity and neurogenesis, and more recently the autophagy hypothesis have been developed to understand the molecular mechanisms of the pathogenesis of depression (Malhi and Mann, 2018; Bar-Yosef et al., 2019). The first observations indicating that autophagy could be implicated in depression were the upregulation of autophagy markers (e.g., beclin 1 and LC3II) and activation of the autophagy signaling pathway upon treatment with antidepressants (Jia and Le, 2015; Gassen and Rein, 2019). In patients with depression, high circulating levels of beclin 1, phosphorylated Akt, and LC3II/LC3I predicted improved clinical antidepressant effect suggesting that antidepressants stimulate macroautophagy induction and autophagosome formation (Rein, 2019). Also, gene expression studies revealed higher expression of autophagy-related genes in blood samples from patients with depression compared with healthy controls (Zhang et al., 2020). Other studies have shown a positive correlation between the expression of autophagy initiators such as beclin 1 and clinical treatment effectiveness (Zhang et al., 2020). Further evidence that suggested the participation of autophagy in depression was the effect of antidepressants on the co-chaperone FKBP51, a regulator of the NR3C1-glucocorticoid receptor involved in the stress response and inhibitor of Akt1 that in turn regulates the activity of beclin 1 in the autophagy signaling pathway (Gassen et al., 2015; Jia and Le, 2015). FKBP51 acts synergically with antidepressants by binding to beclin 1 leading to its phosphorylation which activates the autophagy machinery (Jia and Le, 2015). In animal models of depression, a decrease in autophagy regulators such as Ulk1, AMPK, LC3II/LC3I and an increase in p62 and phosphorylated mTOR suggest reduced autophagy activation (Pierone et al., 2020).

### 7.1. Macroautophagy in depression

Although the exact autophagy-modulating mechanisms of antidepressants have not been fully elucidated, recent evidence shows that macroautophagy induction occurs through antidepressant treatment (Rein, 2019). For instance, fluoxetine and amitriptyline cause the accumulation of sphingomyelin in the lysosomes and ceramide in the endoplasmic reticulum (Rein, 2019). Subsequently, these molecules stimulate the macroautophagy machinery at the initiation phase through the activation of protein phosphatase 2A, Ulk1, beclin 1, and LC3B (Rein, 2019). Other studies have demonstrated a reduction in autophagy markers after induction of depressive-like behavior such as beclin 1, LC3II, and LC3I suggesting dysfunctional mechanisms in the induction and autophagosome formation stages (Pierone et al., 2020). Furthermore, the Hsp90 cochaperone FKBP5, a regulator of the NR3C1/glucocorticoid receptor, has been associated with antidepressant treatment response through the inhibition of Akt, which controls the activity of beclin 1 leading to increased levels of beclin 1, LC3II, LC3I, and Atg12 (Gassen et al., 2015). A positive correlation between the levels of FKBP5 and beclin 1, LC3II, LC3I, and Atg12 and a negative correlation with the phosphorylation of Akt1 suggesting that FKBP5 acts as an autophagy inducer (Gassen et al., 2015). To date, evidence on autophagy regulation in depression shows conflicting results (Zhang et al., 2020) suggesting that the mechanism to maintain the autophagy balance in depression is a dynamic process. For instance, depression-like behavior induced by chronic unpredictable mild stress could activate or inhibit the macroautophagy pathway through beclin 1 and LC3 (Zhang et al., 2020). In other animal models of depression, these macroautophagy markers may behave differently. Upregulation of beclin 1 and LC3 have been observed in depression-like behavior induced by electroconvulsive shot and downregulation occurs in pain-, bacterial endotoxin lipopolysaccharide-, and maternal separation-induced depression-like behavior (Pierone et al., 2020; Zhang et al., 2020). The conflicting results suggest that the autophagic response varies depending on the different stimuli and treatments in depression (Zhang et al., 2020). At the autolysosome level, the number and size of autolysosomes were higher after induction of depression-like behavior by chronic unpredictable mild stress compared to the treatment with serotonin selective reuptake inhibitors (Zhang et al., 2020).

### 7.2. Microautophagy in depression

The potential role of microautophagy in the pathogenesis of depression remains elusive as there are not many studies focused on microautophagy and depression. Glucocorticoids are a class of steroid hormones that are synthesized and secreted in response to stress (Chan et al., 2017). Notably, glucocorticoids are reported to be involved in the pathogenesis of depression by inhibiting neurogenesis in the hippocampus (Chan et al., 2017). Recently, a study showed that glucocorticoids impair both microautophagy and CMA in primary cultured cortical neurons (Sato et al., 2020). Moreover, another recent study reported that glucocorticoids inhibit the degradation of synaptic vesicular proteins in an ESCRT-dependent manner (Sheehan et al., 2016). These findings indicate that glucocorticoid-induced impairment of microautophagy plays an indispensable role in the pathogenesis of depression.

### 7.3. Chaperone-mediated autophagy in depression

The influence of hormonal changes as an important risk factor in depressive-like behavior is starting to be understood. Data suggest that depression is an abnormal brain response to normal hormone changes (Nogrady, 2022). One of the hormones implicated is glucocorticoids, which are released by the adrenal cortex for the regulation of various physiological functions. Chronic stress upregulates glucocorticoid levels, causing degeneration of hippocampal neurons in the central nervous system and mental disorders such as depression (Conrad, 2008). *In vitro*, it was observed that glucocorticoids impaired CMA and microautophagy (Sato et al., 2020). Following chronic treatment of human-derived embryonic kidney cell line (AD293 cells) and primary cultured rat cortical neurons with dexamethasone, a potent glucocorticoid receptor agonist, it was found that CMA activity declined due to a decrease in lysosomal Hsc70 (Sato et al., 2020). Since glucocorticoids are involved in depression, understanding the impact of these agents on CMA activity in neurons can provide an indirect clue to the CMA mechanisms. Also, an endoplasmic reticulum (ER)-stress model of depression was generated recently by hippocampal injection of the antibiotic tunicamycin, which blocks protein glycosylation to induce ER stress (Yuan et al., 2023). Behavioral tests result from the open field test, and forced swimming test in the tunicamycin-injected rats revealed depressive and anxiety-like phenotypes within 8 days. It was determined that synaptic and CMA-protein levels were significantly reduced in the hippocampus of the rats, indicating that these pathways played an important role in the ER-stress depressive rats (Yuan et al., 2023). The disruption of ER homeostasis induces the accumulation of unfolded/misfolded proteins, which in turn causes ER stress (Sicari et al., 2020). Hence, the activation of CMA is a possible defense mechanism for restoring ER homeostasis (Sicari et al., 2020). The evidence backs altered neuronal proteostasis (i.e., protein homeostasis) as a core mechanism underlying the effects of physiological stress on synaptic plasticity (Yang et al., 2014; Martinelli et al., 2018). A terrified-sound stress induced proteomic changes in adult male rat hippocampus (Yang et al., 2014). Similarly, mimicking acute physiological stress in SH-SY5Y cells with a synthetic glucocorticoid receptor agonist was found to alter CMA activity and the levels of synaptic proteins (Martinelli et al., 2018). Expectedly, the knockdown of LAMP2A or the Hsp90 co-chaperone FKBP51 increased the stability of CMA target proteins relevant to synaptic plasticity (Martinelli et al., 2018). These data highlight the significance of different forms of stress on synaptic plasticity and neural activity and reveal the pivotal role of CMA in regulating the neuronal protein changes underlying depressive-like behaviors. Thus, the changes in the expression of chaperones (including Hsc70) and LAMP2A under physiological stress modulate the CMA activity in the central nervous system.

### 7.4. Autophagy *in vitro* methods in depression research

To study the role of autophagy in depression, several *in vitro* models have been developed and a list of approaches is shown in Table 5. Cell-based platforms use glucocorticoid or chemically-induced long-term depression settings as well as antidepressant treatment to create an environment relevant to depression research.

**Table 5:** *In vitro* autophagy methods in depression research.

Autophagy mechanism	Autophagy stage	Molecular target	Cell line	Application (A), description (D), and readout (R)	References
MA	Autophagy induction, autophagosome formation, and autophagic flux	LC3, Atg13, Atg101, Ulk1, p62, Atg5, Atg16L1E-10, Atg9α, and WIPI2	Primary neuronal cells	**A**: Evaluation of the interplay and interdependence between long-term depression and autophagy. **D**: Long-term depression was chemically induced in neurons. Neurons were immunolabeled against LC3. Cells were treated with the autophagy inhibitor wortmannin which inhibits PI3K and Vasp34, SBI-0206965 that selectively inhibits Ulk1. **R**: (1) Protein expression by immunofluorescence targeting and Western blot targeting LC3, Atg13, Atg101, Atg5, Atg16L1E-10, Atg9α, Ulk1, p62, and WIPI2.	Kallergi et al. (2022)
MA	Autophagy induction	Beclin 1, Akt1, LC3II, LC3I, Atg12, FKBP51	HEK293 cells, mouse embryonic cells, rat primary neurons and astrocytes, peripheral blood mononuclear cells from healthy subjects and patients with depression under dexamethasone treatment	**A**: Identification of the role of a regulator of the glucocorticoid receptor (FKBP51) on autophagy upon antidepressant treatment using cells engineered to knockout KFBP51 and Akt1/2. **D**: Astrocytes were engineered to express GFP-LC3 and treated with the autophagy inhibitor BafA1. HEK293 cells and mouse embryonic fibroblasts were engineered to knockout FKBP51 and Akt1/2 to assess the interaction of FKBP51 with beclin 1. Mouse embryonic cells, HEK293, and astrocytes were treated with different antidepressants (e.g., paroxetine, amitriptyline, and fluoxetine). Cells isolated from patients with depression were treated with fluoxetine. **R**: (1) Protein expression by Western blot targeting beclin 1, p-beclin1, Atg12, LC3II, LC3I, P3K, Akt, pAkt. (2) Protein interaction was evaluated by co-immunoprecipitation (interaction between FKBP51 and beclin 1, and Akt and beclin 1). (3) Autophagic flux was evaluated by confocal microscopy targeting LC3.	Gassen et al. (2014)
MA	Autophagy induction and autophagic flux	LC3II., LC3I, Akt, mTOR, and Atg7	Primary hippocampal neurons	**A**: Identification of macroautophagy regulation by neuronal activity after neuronal stimulation by chemical long-term depression. **D**: Cells were treated with the autophagy inhibitor BafA1 in chemical long-term depression conditions in combination with chemical long-term depression inhibitors. Cells were engineered to knock down the expression of Atg7 and to express EGFP-LC3B. **R**: (1) Protein expression by Western blot targeting LC3, Akt, p-Akt, mTOR, p-mTOR, and Atg7. (2) Quantitation of autophagosomes and lysosomes and (4) cell morphology were conducted by confocal microscopy targeting LC3.	Shehata et al. (2012)
MA and CMA	Induction, autophagic flux, autophagosome maturation	LC3II, LC3I, p62, beclin 1, LAMP2A, Akt, and mTOR	SH-SY5Y and HEK293T cells	**A**: Identifying the effect of an antidepressant on macroautophagy by monitoring protein aggregates in neuronal cells and macroautophagy and CMA markers. **D**: Cells were treated with the antidepressant amitriptyline in combination with the autophagy activator rapamycin and inhibitors MG132 and ammonium chloride. Autophagy markers and the formation of protein aggregates were evaluated. Cells were engineered using the mCherry-GFP-LC3 system to assess autophagic flux. HEK293T cells were engineered to express GFP-Arl8 and SKIP to assess the translocation of the lysosome. Cells were treated with a PI3K inhibitor (LY294002) to evaluate MA induction. **R**: (1) Protein expression by Western blot and immunostaining to identify p62, ubiquitin, LC3II, LC3I, LAMP2A, Akt, p-Akt, p-mTOR, and beclin 1. (2) Lysosome translocation was evaluated by confocal microscopy and Western blot targeting KLC1, SKIP, and GFP.	Kwon et al. (2020)
MiA and CMA		LAMP1, LAMP2A, Hsc70, and MEF2D	AD293 cells and cerebral cortex primary neurons	**A**: Evaluation of the effect of glucocorticoids on CMA and MiA in a human-derived cell line and cerebral cortex primary neurons. **D**: AD293 cells were engineered to express glyceraldehyde 3-phosphate dehydrogenase fused with a HaloTag (GAPDH-HT), a marker for CMA and MiA activity, and to knockdown the expression of LAMP2A. Rapamycin was used to activate microautophagy. Cells were treated with the glucocorticoid receptor agonist dexamethasone to decrease CMA and MiA. **R**: (1) Accumulation of GAPDH-HT was assessed by confocal microscopy. (2) Lysosomal isolation was evaluated using a lysosome enrichment kit. (3) Protein concentration of lysosomal lysates was quantified using a bicinchoninic acid protein assay. (4) Protein expression by Western blot targeting LAMP1, LAMP2A, Hsc70, MEF2D, and the HaLo tag.	Sato et al. (2020)

MA, macroautophagy; MiA, microautophagy; CMA, chaperone-mediated autophagy.

A commonly used cell-based platform involved the chemical induction of long-term depression in primary neurons (Shehata et al., 2012; Kallergi et al., 2022). To assess the interplay and interdependence of long-term depression and macroautophagy, cells under long-term depression chemically induced conditions are immunolabeled against LC3 (Kallergi et al., 2022). After treatment of the cells with autophagy inhibitors such as wortmannin, a PI3K and Vasp34 inhibitor, and SBI-0206965, a Ulk1 selective inhibitor, autophagy induction, autophagosome formation, and flux can be monitored by measuring autophagy mediators such as LC3, Atg13, Atg101, Atg5, Atg16L1E-10, Atg9α, Ulk1, p62, and WIPI2 (Kallergi et al., 2022). The chemically induced long-term depression *in vitro* system can be modified further by engineering the cells to knock down autophagy regulators such as Atg7 and express EGFP-LC3 for the assessment of autophagic flux (Shehata et al., 2012). After treatment with the autophagy inhibitor BafA1, the phosphorylated forms of Akt and mTOR can be detected to monitor autophagy induction and quantitation of autophagosomes and autolysosomes can be monitored by morphological analysis using confocal microscopy targeting LC3 (Shehata et al., 2012). Another platform used to study autophagy in depression-like conditions *in vitro* involves the evaluation of KFBP51, a regulator of the glucocorticoid receptor using HEK293 cells, mouse embryonic cells, primary neurons, and astrocytes, and peripheral blood mononuclear cells from healthy subjects and patients with depression under antidepressant treatment (Gassen et al., 2014). In this depression model, cells were engineered to knockout FKBP51 and Akt1/2 and treated with different antidepressants, including paroxetine, amitriptyline, and fluoxetine, and autophagy induction was assessed by identifying autophagy markers and autophagic flux by monitoring the LC3 fluorescence signal (Gassen et al., 2014). Also, FKBP51-mediated induction of autophagy can be monitored by co-immunoprecipitation to analyze the interaction between beclin 1 and FKBP51 and Akt (Gassen et al., 2014). A novel depression method to explore the effect of glucocorticoids on autophagy uses the marker of CMA and microautophagy activity known as glyceraldehyde 3-phosphate dehydrogenase fused with a HaloTag (GAPDH-HT) in a human-derived cell line (AD293 cells) and cerebral cortex primary neurons (Sato et al., 2020). This system relies on the use of GAPDH-HT as a substrate of the CMA and microautophagy systems, measurement of substrate accumulation by confocal microscopy, and monitoring of CMA and microautophagy markers (Sato et al., 2020). In this model, lysosomes were isolated using a lysosome enrichment kit and the lysosomal content was evaluated by a bicinchoninic acid protein assay (Sato et al., 2020). In *in vitro* models of depression, antidepressants are used to assess their effect on autophagy mechanisms. SH-SY5Y and HEK293T cells engineered with mCherry-GFP-LC3 were subjected to treatment with the antidepressant amitriptyline, the autophagy activator rapamycin, and the autophagy inhibitors MG132 and ammonium chloride followed by the analysis of macroautophagy and CMA markers to monitor the autophagy process at different stages (Kwon et al., 2020).

## 8. Conclusion

Autophagy is an important cellular process associated with the pathophysiology of neurodegenerative diseases and mood disorders. In this narrative review, cell-based models designed to study the role of different mechanisms of autophagy in neurodegenerative disorders and depression were presented and discussed. It is now clear that the advances in *in vitro* systems offer the possibility to manipulate different types of cells to exhibit relevant disease hallmarks and modify the expression of key autophagy players to bring to light the mechanistic relationship between disease biology and autophagy. Furthermore, cell-based models that combine the use of autophagy activators and inhibitors are available to provide helpful insights into the role of aberrant autophagy. The catalog of *in vitro* models applied to autophagy research of neurodegenerative disorders and depression include a repertoire of traditional and novel approaches and techniques. These *in vitro* tools can be applied to explore pathophysiological mechanisms at a molecular level and to screen for potential therapeutic agents and their mechanisms of action. Altogether, the use of *in vitro* methods significantly assists in the identification of promising targets to restore autophagy function which can be of great importance to understanding disease biology and potential therapeutic options in the context of neurodegenerative disorders and depression. This is the first review to compile, discuss, and provide a catalog of traditional and novel *in vitro* models applied to neurodegenerative disorders and depression.

## Author contributions

DS-V designed the framework and conceptualization of the review. DS-V, JL, and SA conducted the literature search. All authors contributed to the manuscript writing, revision of the structure and organization of the information in the manuscript and scientifically, revised, and approved the submission of the manuscript.

## Funding

This work was supported by the Start-up Fund for research assistant professors under the Strategic Hiring Scheme (Grant no. P0041471) from The Hong Kong Polytechnic University, Hong Kong SAR awarded to DS-V.

## Conflict of interest

The authors declare that the research was conducted in the absence of any commercial or financial relationships that could be construed as a potential conflict of interest.

## Publisher’s note

All claims expressed in this article are solely those of the authors and do not necessarily represent those of their affiliated organizations, or those of the publisher, the editors and the reviewers. Any product that may be evaluated in this article, or claim that may be made by its manufacturer, is not guaranteed or endorsed by the publisher.
